# Next-generation chemogenetic inhibition using a brain-permeant non-prescription agent

**DOI:** 10.1038/s41392-026-02865-4

**Published:** 2026-07-03

**Authors:** Steven O. Devenish, Sahil D. Patel, Laura V. Ussingkær, Luiz F. Almeida Silva, Olivia Goff, Amy Richardson, Jesse I. Mobbs, Hariprasad Venugopal, David M. Thal, Dimitri M. Kullmann

**Affiliations:** 1 UCL Queen Square Institute of Neurology, University College London, London, UK; 2 Drug Discovery Biology, Monash Institute of Pharmaceutical Sciences, Monash University, Parkville, Australia; 3 ARC Centre for Cryo-electron Microscopy of Membrane Proteins, Monash Institute of Pharmaceutical Sciences, Monash University, Parkville, Australia; 4 Ramaciotti Centre for Cryo-Electron Microscopy, Monash University, Clayton, Australia

**Keywords:** Cellular neuroscience, Medicinal chemistry, Drug development, Neurological disorders

## Abstract

Chemogenetics allows the controllable manipulation of brain circuits upon delivery of a selective activating ligand, and has been invaluable in dissecting brain circuits underlying many behaviours. The Gαi/o-coupled designer muscarinic receptor hM4Di is an especially versatile tool for on-demand inhibition, and has proven effective not only in fundamental neuroscience but also as a therapeutic transgene in preclinical models of epilepsy and other CNS disorders. Indeed, by placing the circuit modulation under the control of an exogenous ligand, chemogenetics mitigates the potential risk of overdosage intrinsic to viral-vector mediated gene therapy. An obstacle to clinical translation, however, is the absence of an activating ligand with favourable biodistribution and side effect profile. Here we show that mutation of hM4Di at two sites (S85 and Y416) imparts full and potent agonism to the widely used over-the-counter antihistamine diphenhydramine. We complement medium-throughput screening in human embryonic kidney cells with in vitro electrophysiological characterization in neuronal circuits, and reveal the interaction of diphenhydramine with key residues using cryo-electron microscopy. Administration of diphenhydramine to mice expressing the modified receptor in the ventral hippocampus reversibly modulated anxiety-related behaviour and attenuated the severity of chemoconvulsant-induced seizures. We further demonstrate on-demand seizure suppression in a chronic epilepsy model. G protein-coupled Receptors Activated by Non-Prescription Agents (GRANPAs) lower the barrier to clinical translation of a powerful chemogenetic approach to brain circuit manipulation.

## Introduction

Many neuropsychiatric disorders are characterized by abnormal neuronal activity in defined brain regions or circuits. Among these are paroxysmal disorders such as epilepsy, psychosis and primary headaches, but also degenerative diseases such as Parkinson’s and Alzheimer’s, which together represent a major burden to society.^1,2^ Treatment with small molecules that affect neuronal or synaptic signalling is generally limited by off-target effects. Against this background, there has been substantial interest in therapies that can be targeted to neurons that participate in abnormal activity, typically using viral vectors to achieve regional and cell-type specificity. Chemogenetics, in particular, offers the possibility to modulate neuronal and synaptic activity in a defined circuit, and to titrate the therapeutic effect by adjusting the dose of the activating ligand, or even to use the ligand on-demand to abort an impending paroxysm.^3,4^

Of the chemogenetic tools, the muscarinic DREADDs (Designer Receptors Exclusively Activated by Designer Drugs)^5^ have been used the most widely in non-clinical settings, both to reveal the fundamental operation of brain circuits in various species, and as experimental therapies in models of epilepsy, Parkinson’s, Alzheimer’s and other diseases.^6–14^ DREADDs are especially versatile because they can be delivered to specific brain regions using vectors such as recombinant adeno-associated viral particles (AAVs), with promoters designed to bias expression to sub-populations of neurons or other cell types in the brain.^4^ The inhibitory DREADD hM4Di is derived from the human M4 muscarinic (hM4) receptor and has been mutated at two amino acids (Y113C and A203G) to reduce the affinity for its normal endogenous agonist acetylcholine (ACh) and to allow high potency activation by clozapine (CLZ) and a number of structurally related compounds, including its inactive metabolite clozapine-N-oxide (CNO).^5^ Clinical translation of chemogenetics with hM4Di requires the use of an activating ligand with high potency and efficacy, good bioavailability and a favourable side effect profile. Although several ligands satisfy the first two criteria, none are known to be safe in the clinic. CLZ is an FDA/EMA-approved drug used to treat psychosis and mania, but it inhibits a range of G protein-coupled receptors (GPCRs) at sub-micromolar concentrations and can trigger hematological abnormalities calling for close monitoring.^15^ We previously identified another anti-psychotic drug in clinical use, olanzapine (OLZ), as a full and potent activator of hM4Di.^16^ Although it is better tolerated, it retains the ‘dirty’ pharmacological profile of CLZ with a similar propensity for inducing hyperglycemia and weight gain.^17^ Both CLZ and OLZ are effective hM4Di activators in rodent models of epilepsy, with potent anti-seizure activity,^18,19^ but their safety and side effect profile underlines the need to identify alternative chemogenetic activators that can be progressed to the clinic for the treatment of epilepsy and other neurological disorders. Although other potent hM4Di activators have been reported, such as ‘Compound 21’ (C21), JHU37152 and JHU37160, and deschloroclozapine (DCZ),^20–22^ the path to approval for use in humans represents a high risk of failure.

An alternative chemogenetic actuator based on a chimeric ligand-gated chloride channel can be activated by alpha-7 nicotinic receptor ligands,^23^ one of which (varenicline) is in clinical use as an anti-smoking aid. However, this too is not without side effects, and the inhibitory effect relies on the trans-membrane chloride gradient, which can be deranged in certain cell types and pathological conditions.^24,25^

Here we describe an alternative approach to lower the barrier to clinical translation of muscarinic DREADDs. Diphenhydramine (DPH), identified as a weak activator of hM4Di,^16^ is widely used as an over-the-counter mild vestibular sedative to relieve symptoms of allergy, hay fever, motion sickness and the common cold. It shares with CLZ and OLZ H1 antagonism, but does not bind to H2, dopamine or noradrenaline receptors at sub-micromolar concentrations, and has substantially higher Kd values at 5HT receptors.^26^ We performed an extensive hM4Di mutagenesis screen, guided by structural data, to increase the potency and efficacy of DPH while maintaining low basal activity. The optimized receptor, which we denote a G protein-coupled Receptor Activated by a Non-Prescription Agent (GRANPA), shows minimal binding of β-arrestin, implicated in desensitization and non-G protein signalling, when activated by DPH. Using cryogenic electron microscopy (cryo-EM) of ligand- and G protein-bound GRANPA, we identify the residues that permit high-affinity binding of DPH and activation of the receptor. We further show that DPH suppresses the activity of GRANPA-expressing neuronal circuits in vitro. Finally, we show rapid and reversible modulation of circuit function in vivo, including anti-seizure efficacy when expressed in the hippocampus.

## Results

### Mutagenesis of hM4Di confers high potency agonism to DPH

**Fig. 1:**
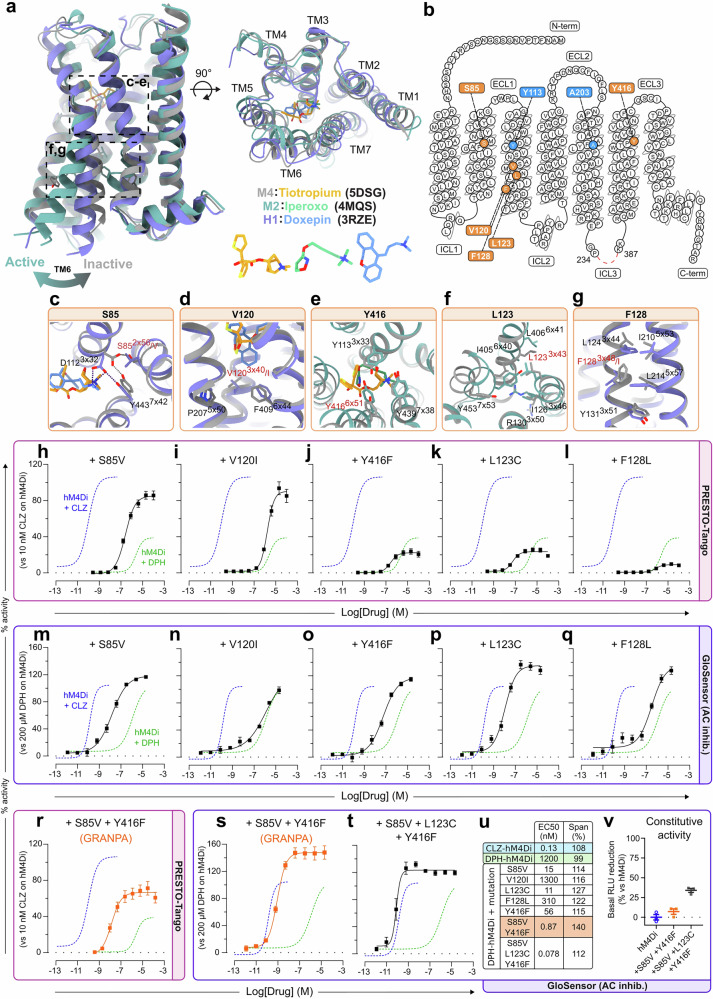
hM4 mutagenesis screen. **a** Aligned crystal structures of hM4 bound to the antagonist tiotropium (5DSG, grey), H1 bound to the antagonist doxepin (3RZE, teal) and hM2 bound to the agonist iperoxo (4MQS, violet). Left: side-view, showing agonist-induced movement of TM6. Dashed boxes highlight the regions zoomed in panels (**c**–**e**), (**f**), and (**g**). Right: top-view, with ligand structures shown below. **b** hM4 snake diagram (without intracellular loop 3) showing the location of Y113 and A203 (blue), which have been mutated to make hM4Di, and the other residues targeted for mutagenesis (orange). **c** D112 (D3x32) engages with the ligand amine via a salt-bridge. An additional hydrogen bond network between D3x32, S85 (S2x56), and Y443 (Y7x42) is present in muscarinic receptors, but is partially disrupted by V2x56 in H1 receptors, resulting in the rotation of D3x32 towards the OBP. **d** Position 3x40 in muscarinic receptors is occupied by a valine residue (V120), but by an isoleucine in H1 receptors, increasing the contact with P5x50 and F6x44 (P207 and F409 in hM4) in adopting active conformations. **e** Y416 (Y6x51), Y113 (Y3x33) and Y439 (Y7x38) together form cation-π interactions with the ligand amine. **f** Breaking the hydrophobic attraction of L123 (L3x43) to I405 and L406 (I6x40 and L6x41) upon agonist binding allows L123 to interact with Y453 (Y7x53), I126 (I3x46) and R130 (R3x50). **g** F128 points into the lipid bilayer and may affect intrahelical movements via hydrophobic interactions with I210 (I5x53) and L214 (L5x57). **h**–**l** Dose–response curves for DPH obtained with the PRESTO-Tango assay for hM4Di + S85V (**h**), hM4Di + V120I (**i**), hM4Di + Y416F (**j**) hM4Di + L123C (**k**) and hM4Di + F128L (**l**) in black. Corresponding curves for DPH and CLZ on unmodified hM4Di are shown as dashed green and blue lines, respectively. Luminescence was normalized to the maximal activation of hM4Di by 10 nM CLZ. **m**–**q** As for (**h**–**l**), but using the GloSensor assay measuring adenylyl cyclase (AC) inhibition, with native β-adrenergic receptors activated by 200 nM isoprenaline. Activity was calculated from the decrease in luminescence from that obtained with isoprenaline alone, and normalized to the response of hM4Di to 200 µM DPH. **r**–**v** Effects of combinations of mutations when activated by DPH on the Tango (**r**) and GloSensor assays (**s**–**u**), and on basal activity on the GloSensor assay (**v**). hM4Di + S85V + Y416F exhibited high potency and efficacy without constitutive activity and is therefore defined as the definitive GRANPA for downstream evaluation (orange). Data are shown as mean ± SEM

We took as a starting point our previous structural similarity screen of molecules related to the full and potent hM4Di agonist C21 (ref. ^20^), which showed that DPH leads to Gβγ-dependent activation of GIRK channels when co-expressed with hM4Di in human embryonic kidney (HEK) cells, albeit with low efficacy when tested at 300 nM.^16^ Although other ligands identified from this screen exhibited higher efficacy, we focused on DPH because of its well-characterised pharmacological profile and availability as an over-the-counter 1st generation antihistamine. DPH is used to treat allergies and motion sickness and is only mildly sedating. We reasoned that further modification of hM4Di, which is itself a mutated hM4 receptor, could result in improved activation by DPH. We aligned hM4 in complex with the antagonist tiotropium (PDB ID: 5DSG)^27^ to doxepin (antagonist)-bound H1 (3RZE)^28^ and iperoxo (agonist)-bound hM2 (4MQS)^29^ (Fig. 1a). As DPH binds with higher affinity to H1 than to hM4,^26^ special attention was given to amino acids that diverge between these two receptors. This led to several candidate residues within the orthosteric binding pocket (OBP) and further down the transduction pathway. Using hM4 for amino acid numbering, these include S85 in trans-membrane helix 2 (TM2), Y113, V120, L123 and F128 in TM3, A203 in TM5, and Y416 in TM6 (Fig. 1b–g). Of these, Y113 and A203, equivalent to Y3x33C and A5x461G by GPCRdb numbering (https://gpcrdb.org) have already been mutated to create the DREADD hM4Di: hM4 + Y113C + A203G (ref. ^5^).

We took advantage of the high signal-to-noise ratio of the β-arrestin based PRESTO-Tango (parallel receptorome expression and screening via transcriptional output, with transcriptional activation following arrestin translocation) assay^30^ to confirm that DPH is a low potency partial agonist of hM4Di when compared to the reference ligand CLZ (Fig. 1h; Supplementary Figs. 1 and 2; Supplementary Tables 1 and 2). Crystal structures 5DSG and 3RZE show that S85 (S2x56) participates in hydrogen bonding with D112 (D3x32), which is absent in H1 receptors (Fig. 1c). In aminergic GPCRs, D3x32 forms a salt bridge with its cognate neurotransmitter, a defining interaction for this class of receptors. As position 2x56 is most commonly occupied by a valine, we hypothesized that the S85V mutation would induce a shift of D112 towards the OBP, allowing for better engagement with the amine in DPH. This prediction was confirmed in the PRESTO-Tango assay, which revealed an increase in the potency (10-fold) and efficacy (130%) of DPH (Fig. 1h).

The ‘microswitch’ residues P5x50, I3x40, F6x44, and W6x48 form conserved ‘PIF’ (proline-isoleucine-phenylalanine) and rotamer toggle switch motifs in aminergic class A GPCRs. These motifs facilitate the rotation and outward movement of TM6 that is required for the receptor to adopt its active conformation.^31^ Muscarinic receptors, however, have a valine instead of an isoleucine at position 3x40, suggesting that this region exhibits greater conformational flexibility^32^ (Fig. 1d). We therefore hypothesized that mutation of this residue in hM4Di (V120, V3x40) to isoleucine would enhance activation by DPH. The V120I substitution had little impact on potency but resulted in an increase in efficacy for DPH (130%) (Fig. 1i).

V2x56 and I3x40 are conserved across virtually all aminergic GPCRs other than muscarinic receptors (Supplementary Figs. 3 and 4). Further amino acid substitutions were therefore guided by the consensus sequence in the aminergic receptor family. Y416 (Y6x51), together with Y113 (Y3x33) and Y439 (Y7x38), contributes to an aromatic lid which forms cation-π interactions with the ligand amine, in addition to directly covering the OBP^29,33^ (Fig. 1e). Y113 was already substituted with cysteine in hM4Di, which provides additional space for the binding of large antagonists in active conformations.^34^ Notably, while Y6x51 is conserved across muscarinic and histaminergic receptors, a phenylalanine is present at this position in all other aminergic GPCRs (Supplementary Fig. 5). The substitution Y416F resulted in an increase in potency for activation of hM4Di by DPH (9-fold) but not efficacy (Fig. 1j).

We next examined residue L123 (L3x43). Mutations of this residue to small (A) or polar (K/N/Q/R/T) amino acids result in constitutive activity in several GPCRs^35,36^ including M1.^33^ This is likely a result of weakening the hydrophobic lock of L3x43, I6x40 and L6x41, the breaking of which precedes the reconfiguration of microswitch residue Y7x53 to interact with L3x43, I3x46 and R3x50, thus increasing and decreasing TM3-TM7 and TM3-TM6 packing, respectively^31^ (Fig. 1f). We confirmed that hM4Di constructs bearing L123 mutations were constitutively active (Supplementary Figs. 6 and 7); however, substitution with C/T/V was accompanied with substantial increases in potency of DPH (42/41/15-fold, respectively) (Fig. 1k; Supplementary Fig. 1).

Finally, F128 (F3x48) was investigated as it is conserved within muscarinic receptors, while a non-aromatic hydrophobic residue occurs at the corresponding position in most other aminergic GPCRs (Supplementary Fig. 4). F128 points out towards the lipid bilayer, only participating in intrahelical as well as hydrophobic interactions with neighbouring TM5 residues I210 (I5x53) and L214 (L5x57) (Fig. 1g). Mutation of F128 to I/L/V led to a small increase in DPH potency (5/3/2-fold, respectively) although with a decrease in efficacy (Fig. 1l, Supplementary Fig. 1). A possible explanation for the increase in potency is that the weakening of TM5 contacts assists in the slight rotation of TM3 to the active conformation.

Since β-arrestin recruitment, which is central to the PRESTO-Tango signalling cascade, is independent from and potentially detrimental to inhibitory Gαi/o signalling, we aimed to validate the conclusions of the mutation screen using a GloSensor™ assay for cAMP. The assay was used to quantify the degree of inhibition of adenylyl cyclase while it was simultaneously activated by isoprenaline acting on Gαs-coupled β-adrenergic receptors endogenous to HEK cells.^37^ This confirmed that S85V, Y416F, L123C and F128L, but not V120I, increased the potency of DPH (81/21/110/4/1-fold, respectively) (Fig. 1m–q).

Finally, we tested combinations of mutations, aiming to uncover cooperative effects on DPH activation (Fig. 1r–t; Supplementary Figs. 1, 2, 8; Supplementary Tables 1, 2, 3). hM4Di carrying both S85V and Y416F mutations exhibited ~1400-fold increase in potency to 870 pM in the GloSensor assay, close to the expected 1700-fold for additive effects of the individual mutations relative to hM4Di (Fig. 1s). There was also a 40% increase in efficacy (measured as ‘span’ or difference between the maximal steady-state and basal activity) (Fig. 1u). This result is consistent with the locations of these mutations on opposite sides of the OBP and in complementary transmembrane domains (TM2 & TM6) to existing hM4Di mutations (TM3 & TM5).

Addition of the L123C or F128L mutations, but not V120I, to hM4Di + S85V + Y416F further improved DPH potency. hM4Di + S85V + L123C + Y416F exhibited an EC50 of 78 pM, a > 15,000-fold shift from hM4Di, marginally surpassing the potency of CLZ-hM4Di on the same assay (130 pM) (Fig. 1t, u; Supplementary Table 3). Some transduction pathway mutations, however, led to varying degrees of constitutive activity, and L123 and F128 mutants were excluded for this reason (Fig. 1v; Supplementary Figs. 6 and 7). In contrast, hM4Di + S85V + Y416F displayed low basal activity, similar to hM4Di, an important property that may contribute to tolerability and durability of circuit manipulation^38^ when expressed in rodent or non-human primate brains^39^ (but see refs. ^40,41^). Therefore, in order to minimize the number of mutations and avoid constitutive activity, hM4Di + S85V + Y416F was chosen as the lead candidate GRANPA for the rest of the study.

### Cryo-EM structure of GRANPA:DPH

**Fig. 2:**
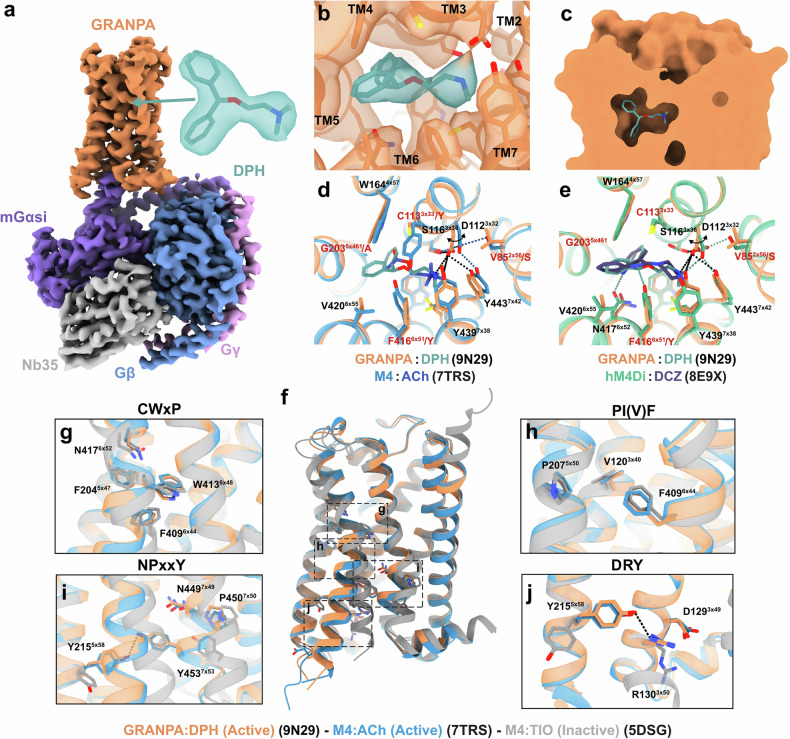
Cryo-EM determination of GRANPA:DPH and conformational changes. **a** Consensus cryo-EM map of GRANPA:mGαsi:DPH determined to 2.55 Å displayed at a level of 0.35. The receptor is shown in orange, mGαsi purple, Gβ1 in blue, Gγ2 in pink, Nb35 in grey and inset showing DPH in teal. **b** Cryo-EM density of the DPH orthosteric binding site. **c** DPH bound to the orthosteric binding pocket. DPH shown in sticks and receptor shown as surface. **d** Comparison of GRANPA:DPH and hM4:ACh (PDB ID = 7TRS). GRANPA receptor is shown as orange sticks and DPH as teal, with hydrogen bonds as black dashes. hM4 is shown as blue sticks, ACh in dark blue and hydrogen bonds in blue. **e** Comparison of GRANPA:DPH with hM4Di:DCZ (PDB ID = 8E9X). hM4Di is shown in green sticks, DCZ as purple and hydrogen bonds as green dashes. Key mutations are labelled red. **f**–**j** Comparison of the common activation motifs of GRANPA:DPH and hM4:ACh active structures with the inactive hM4 tiotropium structure (M4:TIO, PDB ID = 5DSG)

To understand the molecular mechanisms of GRANPA activation, we determined the cryo-EM structure of the active-state GRANPA in complex with G protein transducer (Gαi) and DPH. To enhance the stability of the GRANPA:DPH complex, intracellular loop 3 (ICL3) was truncated as was done for previous hM4 structures,^27^ and a miniature Gαi1 protein (mGαsi1) was incorporated into the C-terminus of the receptor.^42,43^ The complex was co-expressed with Gβ1 and Gγ2 subunits and stabilized further with the nanobody Nb35.^44^ This strategy allowed for the purification of GRANPA-mGαsi-DPH, and the overall complex resolved to 2.6 Å. An additional receptor-focused refinement allowed better interpretation of the receptor at 3.0 Å resolution (Supplementary Fig. 9; Supplementary Table 4). The quality of the cryo-EM density maps was sufficient to model the backbone and sidechains for most of the receptor, G protein, Nb35 and ligand (PDB ID: 9N29; Fig. 2a–e; Supplementary Fig. 10). Compared to the previously determined structures of hM4-Gαi1-ACh (PDB ID: 7TRS)^45^ and hM4Di-mGαo-DCZ (8E9X),^34^ the overall complex had a root mean square deviation (RMSD) of 2.2 Å and 0.8 Å, respectively (Supplementary Fig. 11). The α5 helix region of GRANPA-mGαsi-DPH and hM4-Gαi1-ACh exhibited higher similarity, with an RMSD of 0.67 Å (Supplementary Fig. 11b–d), indicating that they share the same activation motifs (Fig. 2f–j). The differences primarily occur in a subtle, further outward shift of TM6 and a rotation of the G protein, likely owing to the different stabilization strategies used to determine the structures (Supplementary Fig. 11d, e).

Unambiguous cryo-EM density was observed for DPH in the GRANPA OBP (Fig. 2a–c). Molecular recognition of DPH occurs through its three main chemical groups: the two aryl groups, the ether oxygen and the amine tail (Supplementary Fig. 12). The amine group overlays the trimethyl ammonium ion of ACh (Fig. 2d). In the GRANPA:DPH structure, the S85V mutation introduces steric bulk and interrupts a hydrogen bond network between S85 (S2x56), Y443 (Y7x42) and D112 (D3x32), allowing D112 to rotate ~40° towards the OBP and form a hydrogen bond with the amine of DPH. The methyl groups of the amine are surrounded by a hydrophobic pocket formed by Y443, Y439 (Y7x38), and C442 (C7x41). The ether oxygen sits higher in the OBP, towards the extracellular interface, compared to the oxygen in ACh. This region is further surrounded by the hydrophobic F416 (F6x51) on the extracellular side and the toggle switch residue W413 (W6x48) at the base of the OBP. In the structure of hM4Di:DCZ Y416 forms a hydrogen bond to the nitrogen within the middle diazepine ring (Fig. 2e). Loss of this tyrosine residue would suggest impaired activation by these agents,^46^ consistent with the diminished response to CLZ in the PRESTO-Tango assay (although full activity is recovered by addition of S85V in GRANPA) (Supplementary Fig. 2c). DPH has two hydrophobic aryl groups that sit in a hydrophobic pocket formed by G203 (G5x461) and by residues W164 (W4x57), V420 (V6x55), F416 and L190 (in extracellular loop 2). Residues W164 and W413 form π-π stacking interactions that coordinate one of the aryl groups.

### GRANPA pharmacology

Although GRANPA was optimized to exhibit high potency and efficacy for DPH, this does not exclude activation by other ligands. The need to characterize the pharmacological profile of GRANPA is underlined by the finding that, in vivo, CNO activates hM4Di in large part following back-conversion to CLZ,^47^ which has prompted a search for 2nd generation actuators with improved pharmacokinetics and/or potency.^20–22^ Using the TRUPATH assay,^48^ we compared GRANPA activation of Gαi2 signalling by DPH to other clinically approved anti-histamines that are brain-penetrant, structurally similar, and possess a similar binding affinity ratio for muscarinic over histaminergic receptors. The ligands displayed varying degrees of GRANPA activation, with diphenylpyraline possessing slightly lower (albeit nanomolar) potency and efficacy when compared to DPH (Supplementary Fig. 13; Supplementary Table 5). Cyproheptadine also exhibited moderate potency and efficacy. However, DPH displayed the greatest potency and efficacy for GRANPA.

**Fig. 3:**
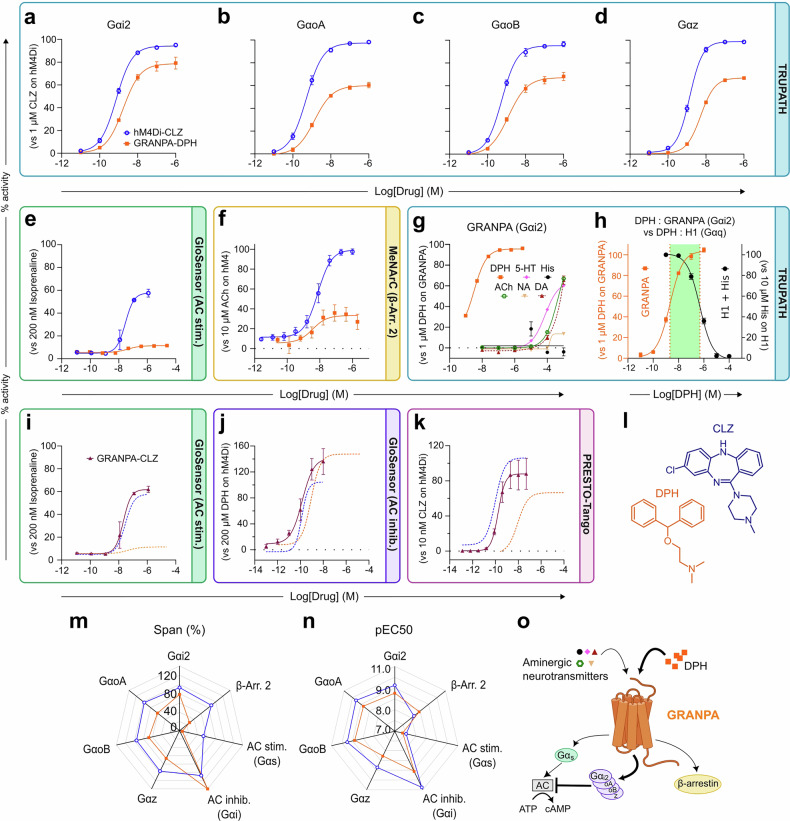
GRANPA-DPH transduction pathways and pharmacological profile. **a**–**d** TRUPATH-based dose–response curves for GRANPA activated by DPH (orange), normalized to hM4Di + 1 µM CLZ (blue), measured for Gαi2 (**a**), GαoA (**b**), GαoB (**c**), and Gαz (**d**). **e** GloSensor-based measurement of adenylyl cyclase (AC) stimulation for GRANPA-DPH and hM4Di-CLZ, normalized to the response to 200 nM isoprenaline. **f** β-Arrestin 2 recruitment measured using the MeNArC assay, normalized to the action of 10 µM ACh on hM4. **g** TRUPATH-based dose–response curve for GRANPA-DPH, and corresponding curves for monoamines (serotonin, histamine, dopamine, and noradrenaline) and ACh. **h** TRUPATH-based assays comparing DPH activation of GRANPA-Gαi2 with inhibition of H1-Gαq in the presence of 10 µM histamine, corresponding to its estimated EC80. **i** GloSensor-based measurement of adenylyl cyclase stimulation normalized to the response to 200 nM isoprenaline, comparing GRANPA-CLZ shown as maroon triangles, to reference curves for hM4Di-CLZ and GRANPA-DPH shown as dashed blue and orange lines, respectively. **j** As for (**i**), but using the GloSensor assay measuring adenylyl cyclase inhibition normalized to the response of hM4Di to 200 µM DPH. **k** As for (**i**), but using the PRESTO-Tango assay normalized to the response of hM4Di to 10 nM CLZ. **l** 2D structures of CLZ (blue) and DPH (orange). DPH represents the prototypical structure of a basic amine and two phenyl groups, whereas CLZ is constrained by tricyclic and piperazine moieties. **m**, **n** Radar plots of Span (**m**) and pEC50 (**n**) comparing the relative activity of GRANPA-DPH and hM4Di-CLZ. **o** Schematic summarizing the pharmacological profile of GRANPA, which is only very weakly activated by neurotransmitters and has low intrinsic activity for Gαs and β-arrestin recruitment (indicated by line thickness). Data are shown as mean ± SEM

Having confirmed DPH as the optimal GRANPA ligand, we examined the downstream transduction pathways by comparing the degree of activation and inhibition of adenylyl cyclase-coupled G protein pathways and β-arrestin (the main desensitization pathway of GPCRs). DPH acting on GRANPA exhibited approximately 3-fold lower potency and 20%-40% lower efficacy than CLZ acting on hM4Di when probed with Gαi2, GαoA, GαoB and Gαz (Fig. 3a–d; Supplementary Table 5). Although this profile implies that the GRANPA-DPH pair does not quite match hM4Di-CLZ as an inhibitory chemogenetic pair, the assay only examines the transduction pathways in isolation. M4 receptors, while primarily coupled to Gαi/o and the related subunit Gαz, can also couple to Gαs.^49^ In comparing the readout of the GloSensor cAMP assay prior to and after the addition of isoprenaline, we probed the balance of stimulation and inhibition of adenylyl cyclase mediated by Gαs and Gαi/o/z natively expressed by HEK cells. cAMP accumulation was substantially greater for hM4Di + CLZ than for GRANPA + DPH (Fig. 3e; Supplementary Fig. 14). Other agonists (ACh, CNO, OLZ and DCZ) also exhibited greater Gαs activation (Supplementary Figs. 15–17, Supplementary Tables 3 and 6), as did DPH acting on GRANPA bearing L123 or F128 substitutions (Supplementary Figs. 8 and 16, Supplementary Tables 3 and 6).

In order to quantify β-arrestin recruitment, we used the MeNArC assay^50^ because this uses an unmodified receptor, unlike the PRESTO-Tango assay which relies on a vasopressin V2 C-terminus fused to the receptor in order to enhance arrestin recruitment (Supplementary Fig. 18). Activation of GRANPA by DPH led to minimal engagement of the β-arrestin pathway (Fig. 3f; Supplementary Fig. 14; Supplementary Table 7).

The incorporation of mutations informed by structural homology with aminergic GPCRs raises the possibility that GRANPA expressed in the brain could bind ambient neurotransmitters, potentially limiting its utility as a chemogenetic tool. We therefore used TRUPATH to assess whether acetylcholine, histamine, dopamine, noradrenaline and serotonin evoke GRANPA-Gαi2 signalling. There was no notable activation of GRANPA by any of these neurotransmitters at concentrations <100 µM (Fig. 3g). Using the GloSensor assay, we further confirmed minimal activation by ACh (Supplementary Fig. 15).

Finally, we related the potency of DPH as a GRANPA agonist to its inhibition of H1 receptors, which underlies its use as a mildly sedating medication to treat symptoms of allergies. We estimated the molecular therapeutic window of DPH by comparing the activation of GRANPA-Gαi2 to the inhibition of H1-Gαq. In the first instance, we constructed an activation curve for histamine acting on H1-Gαq, yielding an EC80 around 10 µM (Supplementary Fig. 19; Supplementary Table 5), which was then used to assess DPH-H1 inhibition. DPH activated GRANPA with ~250x greater potency than it inhibited H1 (Fig. 3h; Supplementary Table 5). Conversely, we estimate that an EC50 concentration at GRANPA (~ 10 nM) yields virtually no inhibition of H1 receptors. This suggests a wide therapeutic window for DPH activating GRANPA over other targets, and, taking into account that DPH does not interact appreciably with other aminergic receptors, implies a low propensity for adverse effects such as sedation at doses that activate Gαi signalling cascades.

CLZ-GRANPA behaves comparably to CLZ-hM4Di across the PRESTO-Tango and GloSensor assays (Fig. 3i–k), implying that the distinct signalling profile of DPH-GRANPA is driven by ligand-specific effects rather than the introduced mutations. Notably, the increased flexibility of DPH relative to the tricyclic CLZ (Fig. 3l) results in a markedly shorter residence time at H1 receptors, a feature that has been linked to biased signalling in other aminergic GPCRs.^51,52^ Taken together, the DPH-GRANPA chemogenetic pair exhibits high potency, minimal signalling via Gαs and β-arrestin, and low propensity for cross-talk with other aminergic signalling cascades (Fig. 3m–o).

### GRANPA allows on-demand inhibition of neuronal network activity and synaptic transmission

**Fig. 4:**
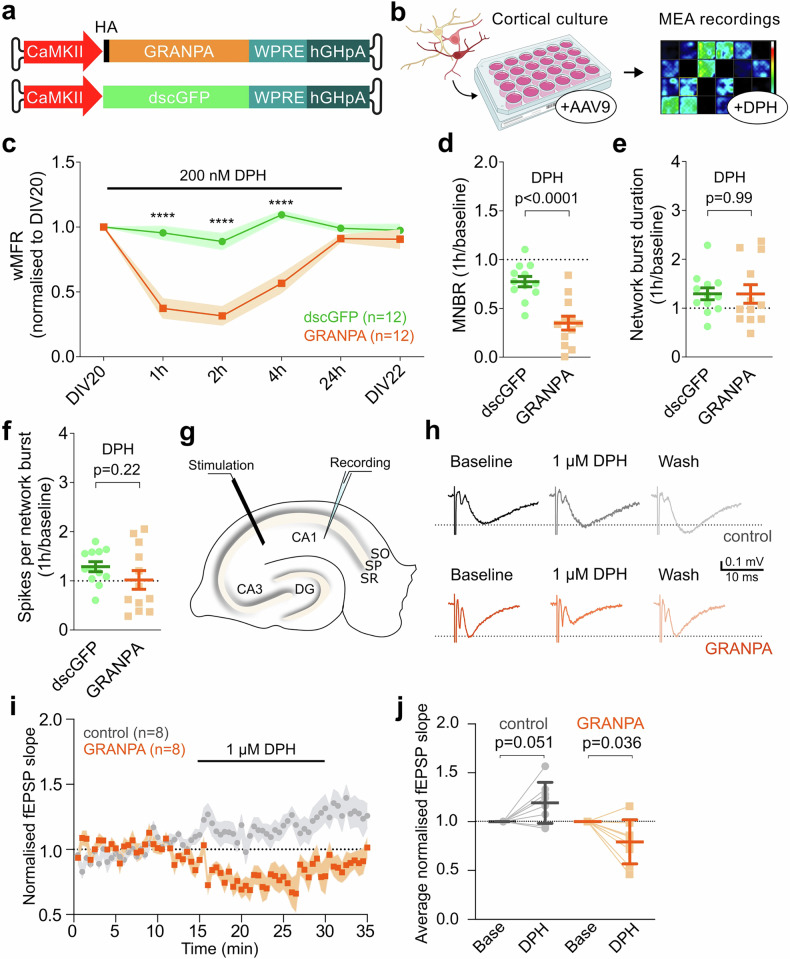
GRANPA-DPH inhibits neuronal circuit activity and neurotransmitter release. **a** Schematic showing design of GRANPA and dscGFP viral vectors under the transcriptional control of a CaMKII promoter. **b** Murine cortical neurons from postnatal day 0 (P0) pups were plated on the electrode fields of multi-electrode arrays and then transduced with AAV9 encoding GRANPA or dscGFP at 6 days in vitro (DIV6). The activity of transduced networks was assessed on DIV20-22 with or without DPH (200 nM). **c** Weighted Mean Firing Rate of transduced cultures at several timepoints following DPH, normalized by pre-DPH baseline values. DPH decreased firing of GRANPA-treated cultures (orange squares, *n* = 12) after exposure for 1 h (1 h), 2 h (2 h) and 4 h (4 h) compared to dscGFP-treated cultures (green circles, *n* = 12) (two-way repeated measures ANOVA with Bonferroni’s multiple comparison test). **d**–**f** The mean network burst rate, network burst duration and number of spikes per network burst of dscGFP and GRANPA networks following DPH treatment for 1 h compared to baseline values (unpaired two-tailed t-tests). Data shown with mean ± SEM. ****: *p* < 0.0001. **g** Schematic showing placement of stimulating and recording electrodes in the SR (stratum radiatum) of hippocampal CA1. Abbreviations: SO, stratum oriens; SP, stratum pyramidale; DG, dentate gyrus. **h** Representative field EPSP (fEPSP) traces recorded from slices taken from uninjected control animals (top) or animals injected with AAV9-CaMKII-GRANPA (bottom) during baseline, 15 min after addition of DPH and 15 min following washout. **i** Average fEPSP slopes, normalized by baseline responses, for WT and GRANPA slices (*n* = 8 slices/group). **j** Quantification of fEPSP slopes during baseline and perfusion of DPH. GRANPA-DPH significantly reduced fEPSP slope (two-way ANOVA with Sidak’s multiple comparison test). Data in (**i**, **j**) shown with mean ± SEM

We asked whether GRANPA can be used for on-demand inhibition of neuronal network activity by expressing the receptor in neuronal cultures grown on multi-electrode arrays (MEAs). We first fused an HA-tag to the N-terminus of GRANPA to allow for immunohistochemical detection,^5^ and then packaged the construct under a CaMKII promoter to bias expression towards excitatory neurons in adeno-associated virus serotype 9 (AAV9) particles (AAV9-CaMKII-HA-GRANPA; Fig. 4a; Supplementary Figs. 20 and 21). Neonatal mouse cortical neurons were plated onto MEAs at a high density to achieve a high level of spontaneous activity.^53^ At 6 days in vitro (DIV), cultures were transduced with either AAV9-CaMKII-HA-GRANPA or a control viral vector expressing green fluorescent protein (AAV9-CaMKII-dscGFP), and network activity was quantified two weeks later (Fig. 4b). At DIV20, prior to the addition of DPH, dscGFP and GRANPA-expressing networks were as viable and active as untransduced networks, suggesting that GRANPA was neither toxic nor tonically active in the absence of agonist (Supplementary Fig. 22a, b). DPH (200 nM) profoundly decreased the weighted mean firing rate (wMFR) of GRANPA- but not dscGFP-treated cultures over time (Fig. 4c). The wMFR of GRANPA-treated cultures decreased to 37% ± 7% (average ± SEM) of the baseline after treatment with DPH for 1 h, with a similar decrease observed in the mean network bursting rate to 35% ± 7% (Fig. 4c, d; Supplementary Fig. 22c). In contrast, the network burst duration and the number of spikes per network burst were unchanged (Fig. 4e, f). After 24-h incubation with DPH, the silencing effect disappeared, and GRANPA-treated cultures were again as active as dscGFP-treated cultures This network effect was also observed with HA-tagged hM4Di in response to CLZ (20 nM) treatment (Supplementary Fig. 22d), and is consistent with homeostatic compensation for activity suppression in neuronal cultures.^54–56^

hM4Di exerts a large part of its inhibitory effects by inhibiting neurotransmitter release at synapses.^6,57^ We asked whether GRANPA has a similar action on glutamate release. AAV9-CaMKII-HA-GRANPA was injected into hippocampi of C57Bl/6J mice, and acute brain slices were prepared three weeks later to examine the effect of DPH on field excitatory postsynaptic potentials (fEPSPs) in stratum radiatum, evoked by Schaffer collateral stimulation (Fig. 4g h). DPH (1 µM) perfusion reduced the fEPSP slope, an effect which was not seen in hippocampal slices prepared from uninjected mice (Fig. 4h–j). The fibre volley amplitude, a proxy measure of the number of axons recruited by the stimulation, was unaffected by DPH (Supplementary Fig. 23a, b), confirming that GRANPA + DPH inhibited glutamatergic transmission rather than presynaptic excitability. Furthermore, the baseline fEPSP slope, normalized by fibre volley amplitude, was similar between GRANPA and control slices (Supplementary Fig. 23c), arguing against basal activity of the receptor in the absence of DPH.

### Hippocampal expression of GRANPA allows on-demand modulation of anxiety and seizures

**Fig. 5:**
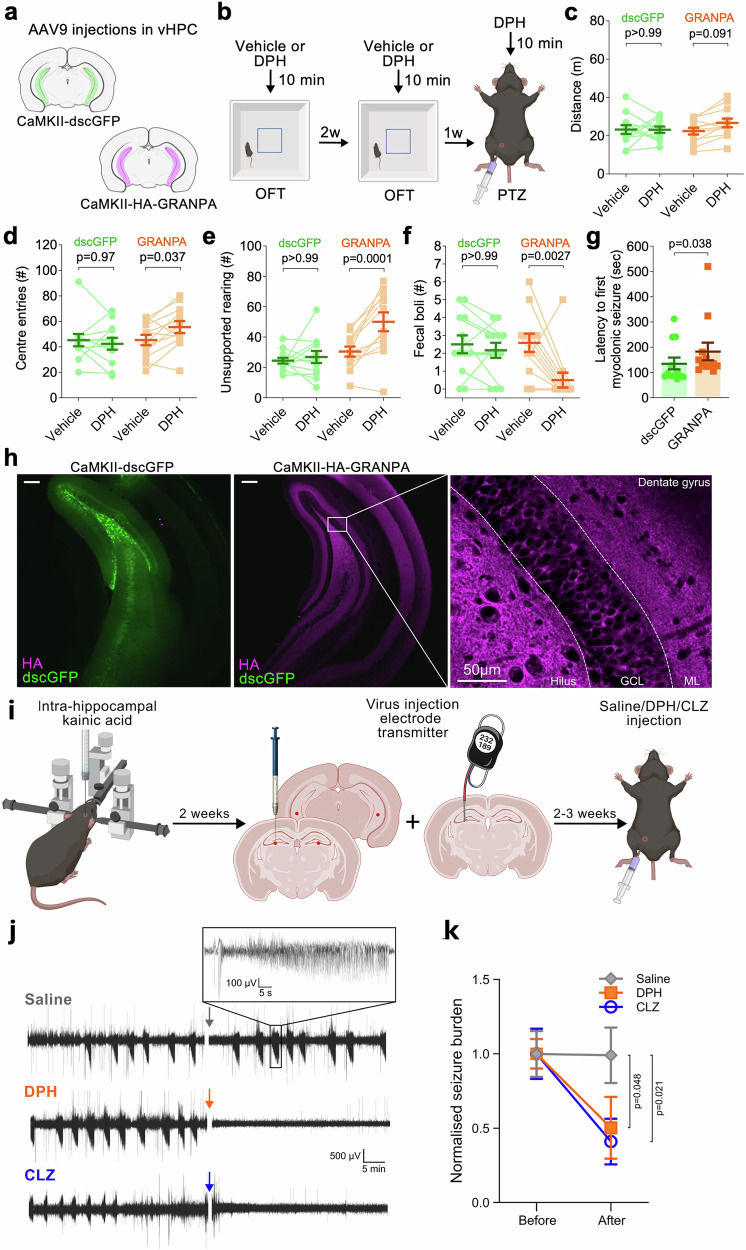
Hippocampal GRANPA-DPH has anxiolytic and anti-seizure effects. **a**, **b** C57BL/6J mice received bilateral adeno-associated viral vector serotype 9 (AAV9) injections in the ventral hippocampi (vHPC). Following three weeks of gene expression, mice were injected with vehicle or DPH, and tested in the open field test (OFT) to quantify anxiety. Two weeks later, the same mice received an intraperitoneal injection of vehicle or DPH in a counterbalanced fashion and were re-tested in the OFT. Mice were subsequently injected with DPH again and then with the chemoconvulsant pentylenetrazol (PTZ) at the end of the study to examine seizure susceptibility. **c** The activity of mice injected with dscGFP (green circles, *n* = 12) or GRANPA (orange squares, *n* = 12) following vehicle and DPH treatment (two-way repeated measures ANOVA with Bonferroni’s multiple comparison test). **d**–**f** Centre entries, unsupported rearing and number of fecal boli following vehicle and DPH treatment (two-way repeated measures ANOVAs with Bonferroni’s multiple comparison test). **g** Latency to the first PTZ-induced myoclonic seizure in GRANPA compared to dscGFP animals following DPH treatment (*n* = 12 mice for each group; two-tailed Mann-Whitney U test). Data in (**c**–**g**) shown with mean ± SEM. **h** Hippocampal slices show the presence of dscGFP in cell bodies of the ventral hippocampus of a dscGFP mouse, whereas HA-tag fluorescence shows widespread membrane expression. Scalebar: 0.2 mm (left, centre) and 50 µm (right). GCL- granule cell layer; ML – molecular layer. **i** Schematic showing the induction of the mesial temporal lobe epilepsy model characterized by frequent focal seizures detected via an intrahippocampal electrode. **j** Sample EEG traces showing seizures before and after injection of saline, DPH or CLZ at time indicated by the arrows. Insert: magnified trace showing characteristic sentinel spike and seizure evolution. **k** Seizure burden, calculated as total time spent in a seizure state during 120 min periods before and after saline, DPH or CLZ, normalized to the pre-injection baseline (*n* = 5). Both DPH and CLZ reduced the seizure burden (two-way ANOVA with Fisher’s LSD), with the main effect on seizure frequency rather than duration. Data are shown as mean ± SEM

Chemo-and optogenetic studies have previously shown that inhibition of the ventral hippocampus has an anxiolytic effect.^58,59^ We therefore expressed either AAV9-CaMKII-HA-GRANPA or AAV9-CaMKII-dscGFP in the ventral hippocampi of C57Bl/6J mice, and tested the effect of DPH on anxiety-related behaviours three weeks later (Fig. 5a). Mice were randomized to intraperitoneal (IP) injection of DPH (1 mg/kg) or vehicle, and then observed in an open field test (OFT). After a further two weeks, mice underwent the same protocol with vehicle and DPH treatment switched around, and with the arena in a different part of the room to enhance environmental novelty (Fig. 5b). Data were collected and analysed blind to the viral vector and drug treatment. While DPH had no effect on the total distance travelled in either group of mice (Fig. 5c), it increased the number of entries into the centre of the arena in GRANPA-expressing mice, but not in dscGFP-expressing control mice (Fig. 5d; Supplementary Fig. 24a, b), consistent with decreased anxiety. DPH activation of GRANPA was also associated with a non-significant trend for reduced thigmotaxis (Supplementary Fig. 24c), a profound increase in unsupported rearing and a reduction in fecal boli (Fig. 5e, f), further confirming an anxiolytic effect.^60,61^ Mice did not show any signs of habituating to the test (Supplementary Fig. 24d–f).

Seizures have been reported as rare consequences of CLZ exposure or DPH overdose,^62,63^ and CLZ has no intrinsic anti-seizure effect in mice.^18^ Nevertheless, we asked if these agonists have anti-seizure effects when GRANPA is expressed in the hippocampi. We initially used the same mice as for the behavioural experiments, and evoked seizures with 50 mg/kg IP pentylenetetrazol (PTZ) injection 10 min after 1 mg/kg DPH injection. The latency to the first myoclonic seizure, characterized by neck or tail jerking, was significantly longer in GRANPA-expressing mice than in dscGFP-expressing control mice (Fig. 5g). GRANPA-expressing mice showed a tendency for a longer latency to seizure generalization, but this fell short of significance (Supplementary Fig. 25). After sacrifice, we confirmed that HA-tagged GRANPA was expressed in the ventral hippocampi (Fig. 5h), in a pattern similar to that of native M4 receptors in rodents.^64^

In order to evaluate the ability to suppress spontaneous, as opposed to evoked, seizures, we turned to a chronic model of mesial temporal lobe epilepsy evoked by kainate injection in the dorsal hippocampus (Fig. 5i). In this model, a period of status epilepticus is followed by a latent period, and then a high frequency of spontaneous focal seizures that can be detected by intrahippocampal electrodes. A mirror focus can also develop, consistent with strong commissural connections in rodents.^65^ In epileptic mice treated with AAV9-CaMKII-HA-GRANPA bilaterally in the dorsal and ventral hippocampi, injection of GRANPA agonists DPH or CLZ (1 mg/kg), but not saline, with analysis blind to injected substance, led to a marked reduction of seizure burden (Fig. 5j, k). In a separate cohort of control animals expressing mCherry in the hippocampus, CLZ had no effect (Supplementary Fig. 26, Supplementary Table 8). The results indicate that GRANPA + DPH may be applicable as an on-demand, and potentially titratable, therapeutic tool for brain circuit disorders.

## Discussion

We describe a novel chemogenetic pair, consisting of DPH as the ligand and GRANPA (hM4Di + S85V + Y416F) as a modified inhibitory muscarinic DREADD. All previously characterized hM4Di activating ligands are close analogues of CLZ, a tricyclic antipsychotic which binds to multiple aminergic GPCRs. DPH, by comparison, is a first generation antihistamine which, while structurally distinct from CLZ, shares a common pharmacophore for binding to H1 and muscarinic receptors. Importantly, the safety profile of DPH far exceeds that of other repurposable DREADD agonists including OLZ,^16^ as demonstrated by its availability in several countries as a non-prescription-agent. Indeed, DPH inhibits muscarinic receptors at concentrations >100-fold higher than its EC50 at GRANPA.

Although the potency of DPH acting on GRANPA does not quite match that of CLZ acting on hM4Di (EC50 0.9 nM and 0.1 nM, respectively), DPH-GRANPA signalling exhibited reduced Gαs stimulation relative to CLZ-hM4Di. Since GPCR-mediated suppression of neurotransmitter release is mediated in part by adenylyl cyclase inhibition,^66^ the relative bias of DPH-GRANPA away from Gαs signalling may compensate for lower overall potency.

In common with most class A GPCRs, muscarinic receptors undergo β-arrestin-mediated termination of G protein signalling.^67^ This process contributes to tolerance in the medical use of opioids and may likewise account for mixed reports of diminished silencing following continuous activation of hM4Di.^68^ In our experiments, DPH-GRANPA demonstrated minimal recruitment of β-arrestin. Notably, the M1/M4 selective agonist xanomeline, recently approved for the treatment of schizophrenia, fails to recruit β-arrestin.^69,70^ This suggests that prolonged G protein signalling via M4 receptors is clinically viable.

The relative contributions of presynaptic and postsynaptic actions of muscarinic DREADDs remain to be determined. Because the mutations inserted in GRANPA are far from cytoplasmic elements that regulate trafficking to different neuronal compartments,^71^ this aspect is unlikely to differ substantially from hM4Di and unmodified M4 receptors. Similarly, the mutations are unlikely to affect membrane expression of GRANPA, which can alter the apparent efficacy and potency of the receptor, other than through a possible reduction of β-arrestin mediated internalization.

Direct comparison of DPH dosage in mice and humans is not straightforward. Although DPH undergoes active transport into the CSF in several species^72^ it is cleared far more rapidly in rodents than in humans.^73,74^ Nevertheless, at 1 mg/kg, a dose sufficient to exert anxiolytic and anti-seizure effects when GRANPA was expressed in the ventral hippocampus, DPH-treated mice exhibited a non-significant trend for increased locomotion, arguing against a sedating effect. This dose is ~10-fold lower than the dose required to reduce spontaneous locomotion in mice,^75^ and ~100-fold below the LD50.^76^ Whether N-desmethyldiphenhydramine and other metabolites of DPH contribute to its action on GRANPA remains to be determined. We speculate that loss of an N-methyl group could decrease potency, by analogy with the 42-fold lower affinity of deschloro-N-desmethylclozapine (‘compound 21’) than deschloroclozapine at hM4Di.^22^

In the present study we have demonstrated an anxiolytic effect of DPH following expression of GRANPA in the ventral hippocampus. This is consistent with connectivity of this structure with other nuclei implicated in context-dependent fear (amygdala), behavioural avoidance (medial prefrontal cortex), and fight-or-flight decisions (lateral hypothalamus).^77^ The ventral hippocampus is also an important seizure generator in rodent models of temporal lobe epilepsy,^78^ which is the commonest subtype of focal epilepsy encountered in clinical practice. In the present study we explored the ability of DPH to suppress seizures in a mouse model of temporal lobe epilepsy where GRANPA was expressed bilaterally in the ventral hippocampi. This experimental design takes into account the profuse commissural connection between ventral hippocampi that exist in rodents, which allow mirror seizure foci to develop rapidly during epileptogenesis.^65^ Although this connection is very sparse in humans, unlike the dorsal hippocampal commissure,^79^ bilateral seizure foci occur in up to 30% of people diagnosed with pharmacoresistant temporal lobe epilepsy, and represent a major unmet therapeutic need since surgical ablation represents an unacceptable risk of irreversible cognitive decline.^80^ We propose chemogenetic treatment with DPH-GRANPA as a candidate therapy in such cases, as well as in other subtypes of epilepsy where precise adjustment of the therapeutic effect is required for an optimal trade-off of seizure suppression and impairment of normal function.

Taken together, although hM4Di remains a powerful tool for basic research, DPH-GRANPA is a candidate chemogenetic pair amenable to clinical translation in epilepsy and other diseases of circuit hyperactivity. In general, chemogenetics allows the regional and cell-type specificity of gene therapy to be combined with the ability to titrate the therapeutic effect, which cannot easily be achieved by adjusting viral vector dose. Subject to further validation, DPH-GRANPA potentially allows chemogenetic inhibition to be achieved relatively safely, whether as a continuous treatment or on-demand, for instance to rescue patients experiencing crescendo seizures,^81^ worsening trigeminal autonomic cephalalgia^82^ or relapse of psychosis.^83^

## Materials and methods

### Molecular biology

Site-directed mutagenesis was performed using the QuikChange II XL site-directed mutagenesis kit (Agilent, Fisher Scientific cat. no. NC9045762) or the Q5 Site-Directed mutagenesis kit (NEB, cat. no. E0554S), and verified by Sanger sequencing. Plasmids for transfection were purified with the NucleoBond^TM^ Xtra Midi Plus Endotoxin Free kit (Macherey-Nagel™, Fisher Scientific, 740422.50).

### Cell culture

HEK-293T cells were maintained in Dulbeco’s modified Eagle medium (DMEM) with high glucose, pyruvate and GlutaMAX^TM^ (Gibco^TM^, Thermo Fisher Scientific, cat. no. 31966047), supplemented with 10% (v/v) Foetal Bovine Serum (FBS), 50 units/mL penicillin and 50 µg/mL streptomycin (Thermo Fisher Scientific, cat. no. 15070063). Cells were maintained between 10% and 90% confluency at 37 °C in the presence of 5% CO_2_. HTLA cells^84^ (a HEK293 cell line stably expressing a tTA-dependent luciferase reporter and a β-arrestin2-TEV fusion gene) were maintained similarly, with the addition of 2 µg/ml puromycin (Thermo Fisher, cat. no. A1113803) and 100 μg/ml hygromycin B (Thermo Fisher, cat. no. 10687010).

### PRESTO-Tango assay

The day prior to transfection, HTLA cells were plated in T-25 flasks (900,000 each) with 4 mL complete medium. Flasks at 50–70% confluency were transfected with 4 µg of the receptor containing Tango plasmid DNA (Addgene plasmid #66251), prepared in 400 µL Opti-MEM^TM^ (Gibco^TM^, Thermo Fisher Scientific, cat. no. 31985062) with 12 µL TurboFect^TM^ transfection reagent (Thermo Scientific™, Thermo Fisher Scientific, cat. no. R0532). The following day, drug dilutions were prepared in Opti-MEM and 37.5 µL was added to each well of a poly-D-lysine treated (Gibco™, Thermo Fisher Scientific, cat. no. A3890401), white 96-well cell culture plate (Greiner Bio-One CELLSTAR, cat. no. 655083). The transfected cells were then detached with trypsin solution and 37.5 µL was re-seeded in Opti-MEM at a density of ~80,000 cells per well.

Plates were removed from the incubator the next day and left to equilibrate for 20 min at RT, alongside the Glo Lysis buffer (Promega, cat. no. E2661) and ONE-Glo^TM^ luciferase (Promega, cat. no. E6110). For each plate, 1 ml of ONE-Glo^TM^ luciferase was diluted in 7 ml of Glo Lysis buffer, with 70 μL added per well. After a 5 min incubation at RT on an orbital shaker, the luminescence was counted using a Tristar 5 Multimode Microplate Reader (0.5 s integration time; Berthold Technologies). Relative luminescence units (RLU) were normalized to the fold change of 10 nM CLZ on reference wells expressing hM4Di (8 per plate), and analysed using “log(agonist) vs. response -- Variable slope (four parameters)” in GraphPad Prism 10.5.0. All dose–response curves were run with 2 technical replicates, with each N (≥ 3) representing an individually transfected T-25 flask.

### GloSensor assay

The day prior to transfection, HEK-293T cells were plated in T-25 flasks (900,000 each) with 4 mL complete medium. Flasks at 50–70% confluency were co-transfected with 2 µg of the receptor of interest in pcDNA5 and 2 µg pGloSensor-22F, prepared in 400 µL Opti-MEM with 12 µL TurboFect transfection reagent. The following day, transfected cells were detached with Accutase, triturated in DMEM supplemented with 1% (v/v) dialyzed FBS, and 100 µL plated per well of a poly-D-lysine treated, white 96-well clear-bottom cell culture plate (Greiner cat no 655983) (~60,000 cells). Plates were incubated overnight, and wells were washed once with 150 µL assay buffer (1X Hank’s balanced salt solution (HBSS; Gibco^TM^, Thermo Fisher Scientific cat. no. 14175137) complete with 1 mM MgCl_2_, 1.2 mM CaCl_2_ and 20 mM HEPES pH 7.3), followed by the addition of 80 µL assay buffer with 2.4% (v/v) Promega GloSensor assay reagent (Promega, cat. no. E1291). After 60 min at RT, white backing was applied, and 20 µL of serially diluted drug, buffer, or control DPH was added to wells, with plates placed on an orbital shaker to incubate for 15 min. RLU was measured using a Tristar 5 with 1 s integration time, providing adenylyl cyclase stimulation as readout. 20 µL of 1.2 µM isoprenaline (6x solution, final concentration 200 nM) was added and the plate returned to the shaker for an additional 15 min prior to re-measurement for determination of adenylyl cyclase inhibition.

All dose–response curves were run with 2 technical replicates, with adenylyl cyclase inhibition calculated as the decrease in luminescence relative to wells from the same transfected flask treated with buffer instead of drug (2 per replicate). The % inhibition was normalized to the inhibition by 200 µM DPH on an individually transfected flask of hM4Di (4 wells each of buffer+isoprenaline and DPH+isoprenaline per plate). The % stimulation was normalized to the 4 control wells of hM4Di treated with buffer+isoprenaline. All data was analysed using “log(agonist) vs. response -- Variable slope (four parameters)” in GraphPad Prism 10.5.0, with each N (≥3) representing an individually transfected T-25 flask. In some instances where a curve could not reliably be fit, the Hillslope was constrained to 1.

A separate experiment was conducted to determine basal adenylyl cyclase inhibition by certain constructs. A 96-well plate was run with 3 biological replicates (each with 4 technical replicates) of each receptor treated with 200 nM isoprenaline. Constructs were staggered across the plate to reduce edge effects. The % reduction was calculated relative to the mean RLU of hM4Di. A one-way ANOVA with Dunnett’s test was performed in GraphPad Prism 10.5.0.

### TRUPATH assay

The day prior to transfection, HEK-293T cells were plated in T-25 flasks (900,000 each) with 4 mL complete medium. Flasks at 50–70% confluency were co-transfected with the receptor of interest in pcDNA5, along with TRUPATH Gα-RLuc, Gβ and Gγ-GFP2 plasmids (4 µg total DNA in a 1:1:1:1 ratio; Addgene kit #1000000163), prepared in 400 µL Opti-MEM^TM^ with 12 µL TurboFect^TM^ transfection reagent. The following day, transfected cells were detached with trypsin, and 50 µL was re-seeded in Opti-MEM^TM^ at a density of 40,000 cells per well onto poly-D-lysine treated, white 96-well cell culture plates.

The following day, wells were aspirated and washed twice with assay buffer (1X HBSS complete with 1 mM MgCl_2_, 1.2 mM CaCl_2_ and 20 mM HEPES pH 7.3). Each well was incubated with 7.5 µM Prolume Purple (Nanolight Technology, Cat. No. 369) in 60 µL assay buffer for 5 min before the addition of 30 µL of serially diluted drugs. Plates were incubated for a further 5 min, followed by loading onto a Tristar 5 with 395 nm and 510 nm emission filters reads performed with a 1 s integration time. The plates were read consecutively four times with measurements from the third reading used for all analyses (15 min after drug addition). The BRET2 ratio was calculated as the ratio of GFP emission to Rluc8 emission. The ΔBRET2 was calculated as the change in BRET ratio relative to buffer only wells. The ΔBRET2 ratio was then normalized to an independent control receptor + drug, and analysed using “log(agonist) vs. response -- Variable slope (four parameters)” in GraphPad Prism 10.5.0. All dose–response curves were run with 2 technical replicates, with each N (≥ 3) representing an individually transfected T-25 flask.

### MeNArC assay

The day prior to transfection, HEK-293T cells were plated in T-25 flasks (900,000 each) with 4 mL complete medium. Flasks at 50–70% confluency were co-transfected with 0.25 µg of the receptor of interest in pcDNA5, 0.1 µg MeNArC (polycistronic vector),^50^ and 2.0 µg GRK2 (Addgene plasmid #23835 which was subsequently cloned into a pcDNA3.1 backbone), prepared in 400 Opti-MEM^TM^ with 12 µL TurboFect^TM^ transfection reagent. The transfected cells were then detached with trypsin solution and 50 µL was re-seeded in Opti-MEM at a density of ~40,000 cells per well.

The next day, wells were aspirated and washed with assay buffer (1X HBSS complete with 1 mM MgCl_2_, 1.2 mM CaCl_2_ and 20 mM HEPES pH 7.3). Each well was incubated with 7.5 µM furimazine in 45 µL assay buffer for 5 min before the addition of 30 µL of serially diluted drugs. Plates were incubated for a further 5 min, followed by loading onto a Tristar 5 with luminescence emissions read every 5 min for 4 reads (0.5 s integration time). The 15 min read was plotted relative to the buffer only wells and normalized to an independent control receptor, followed by analysis using “log(agonist) vs. response -- Variable slope (four parameters)” in GraphPad Prism 10.5.0. In some instances where a curve could not reliably be fit, the Hillslope was constrained to 1. All dose–response curves were run with 2 technical replicates, with each N ( ≥ 3) representing an individually transfected T-25 flask.

### Purification of GRANPA-mGαsi-DPH complex

The full-length human M4 muscarinic receptor gene was modified to include the 4 mutations (S85V^2x56^, Y416F^6x51^, Y113C^3x33^ and A203G^5x461^) to make the GRANPA receptor. It was further modified to include an N-terminal hemagglutinin (HA) signal sequence and FLAG-tag, and C-terminal 8xhistadine tag (8xHis), GGGS linker, 3C protease cleavage site, and mini-Gsi (mGαsi).^85^ The resulting FLAG-GRANPA-mGαsi construct was cloned into the pFastBac vector (Invitrogen) for insect cell expression.

FLAG-GRANPA-mGαsi and Gβ1γ2 were co-expressed in Tni insect cells (Expression Systems), using the Bac-to-bac baculovirus expression system (Invitrogen). The Tni cells were grown to a density of 4 × 10^6^ cells/ml in ESF 291 serum-free media (Expression Systems) and infected with a 4:1 ratio of FLAG-GRANPA-mGαsI:Gβ1γ2 baculoviruses. Cells were incubated for approximately 48–60 h at 27 °C, before harvesting by centrifugation (6000 × *g*, 20 min, 4 °C), and cell pellets stored at −80 °C.

For the purification of FLAG-GRANPA-mGαsi complex, cell pellets were thawed and resuspended in lysis buffer (20 mM HEPES pH 7.4, 5 mM MgCl_2_) with protease inhibitors (0.2 mM phenylmethylsulfonyl fluoride (PMSF), 5 µg/ml leupeptin, 5 µg/ml soybean trypsin inhibitor and 1 mM benzamidine hydrochloride), 500 units benzonase and 1 μM DPH. The resuspension was stirred at RT for 15 min to allow for lysis before centrifugation at 30,000 × *g* for 20 min at 4 °C. The pellet was resuspended in solubilization buffer (20 mM HEPES pH 7.4, 100 mM NaCl, 5 mM MgCl_2_, 5 mM CaCl_2_, 0.5% lauryl maltose neopentyl glycol (LMNG), 0.03% cholesteryl hemisuccinate (CHS)) with protease inhibitors (0.2 mM PMSF, 5 µg/ml leupeptin, 5 µg/ml soybean trypsin inhibitor and 1 mM benzamidine hydrochloride), 500 units benzonase. The resuspended pellet was Dounce homogenized and complex formation initiated with the addition of 10 μM DPH, 1 mg Nb35 and 25 mU/ml apyrase. The complex was incubated with stirring for 2 h at 4 °C before centrifugation at 30,000 × *g* for 30 min at 4 °C to remove insoluble material. The complex was then bound to M1 FLAG-affinity resin by gravity flow before washing with wash buffer (20 mM HEPES pH 7.4, 100 mM NaCl, 5 mM MgCl_2_, 5 mM CaCl_2_, 0.01% LMNG, 0.0006% CHS and 1 μM DPH). Complex was eluted with elution buffer (20 mM HEPES pH 7.4, 100 mM NaCl, 5 mM MgCl_2_, 0.01% LMNG, 0.0006% CHS, 1 μM DPH, 10 mM EGTA and 0.1 mg/ml FLAG peptide) before concentration in an Amicon Ultra-15 100 kDa molecular mass cut-off centrifugal filter unit (Millipore). The complex was further purified by size exclusion chromatography on a Superdex 200 Increase 10/300 GL (Cytiva) in 20 mM HEPES pH 7.4, 100 mM NaCl, 5 mM MgCl_2_, 0.01% LMNG, 0.0006% CHS and 1 μM DPH. Final fractions were assessed by SDS-PAGE, pooled, concentrated to 10 mg/ml and flash frozen in liquid nitrogen before storage at −80 °C.

### Grid preparation and imaging

3 µL of sample was applied to glow-discharged (15 mA, 180 s) UltrAufoil R1.2/1.3 300 mesh holey grid (Quantifoil) and were frozen in liquid ethane using a Vitrobot mark IV (Thermo Fisher Scientific) at 100% humidity and 4 °C with a blot time of 2 s and blot force of 12. Data were collected using a G1 Titan Krios microscope (Thermo Fisher Scientific) equipped with S-FEG, a BioQuantum energy filter and K3 detector (Gatan). The Krios was operated at an accelerating voltage of 300 kV with a 50 μm C2 aperture, 100 μm objective aperture inserted, and zero-loss filtering (10 eV slit width), at 105kx magnification in nanoprobe EFTEM mode. Data were collected using aberration-free image shift (AFIS) with Thermo Fisher EPU software.

### Cryo-EM data processing

7091 movies collected at 0.82 Å/pix were motion corrected using RELION v5.0 implementation of MotionCor2, output as float 16,^86,87^ and contrast transfer function (CTF) parameters were estimated using CTFfind 4.1.^88^ 6554 micrographs with a max CTF resolution of 5 Å were carried forward for processing. 2.9 M particles were picked from motion-corrected micrographs using the general model of Cryolo^89^ and extracted within RELION v5.0 (60 pix, 4.92 Å/pix). Particle stacks were imported to CryoSparc V4.1.2 for 2D classification and ab initio model generation. Heterogeneous refinement was performed with an initial model and 1 junk model. Full sized particles were re-extracted with RELION v5.0 (360 pix, 0.82 Å/pix) and subjected subsequent rounds of 2D classification and heterogeneous refinement in cryoSPARC v4.1.2. A set of particles (189 K) was subjected to Bayesian Polishing in RELION v5.0 and a final non-uniform refinement was performed with CTF refinement. Finally, to improve the quality of the receptor, a mask was generated, and a local refinement was performed.

### AAV9 vector preparation

Gene therapy constructs CaMKII-dscGFP-WPRE-hGHpA and CaMKII-HA-GRANPA-WPRE-hGHpA were packaged into AAV9 particles as previously described.^53^ The concentration of DNase I-resistant viral genomes was determined by qPCR using SYBR green technology (Bio-Rad, 1725120) and primers complementary to the AAV2 ITRs.^90^ Briefly, an optical plate was loaded with serial dilutions of DNase I-treated AAV9 vector preparations alongside serial dilutions of a TdTomato plasmid of known concentration (Addgene plasmid #59462). Following the addition of reagents listed above, thermocycling commenced on a QuantStudio system (Applied Biosystems) using the following settings: one cycle of 98 °C for 3 min followed by 40 cycles of amplification (98 °C for 15 s, then 58 °C for 30 s) and a melt curve. A standard curve was generated by linear regression of average Ct values versus the log of known plasmid copy number per mL, which was used to determine the number of viral vector genomes per mL for AAV9 samples. The protein composition of AAV9 samples was determined using SDS-PAGE. Briefly, AAV9 samples were denatured in LDS buffer (ThermoFisher, NP0007) at 97 °C for 5 min and then loaded onto a Bis-Tris gel (Invitrogen, NP0321BOX) alongside a protein ladder (ThermoFisher, 26619). The gel was run in MOPS SDS buffer (Invitrogen, NP0001) at 70 mV for 15 min, followed by 110 mV for 1 h. After incubation with a fixing solution made from methanol (50%), dH_2_O (43%) and glacial acetic acid (7%) for 1 h shaking, the proteins were stained with SYPRO Ruby (ThermoFisher, S12000) O/N shaking, washed with dH_2_O three times and imaged with a Gel Doc XR molecular imager using Image Lab software (Bio-Rad). The percentages of empty and full capsids were estimated in viral vector samples diluted to around 10^11^ vg/mL in PBS using a SamuxMP mass photometer (Refeyn). Counts for empty and full capsids were given by the nearest peak to 3.7 MDa and 5.0 MDa, respectively. Particles with masses of <2 MDa were defined as protein impurities, and particles with a mass intermediate between empty and full populations were classified as partially full or ambiguous.

### Animals

Black C57BL/6J male mice procured from Charles River UK were co-housed in individually-ventilated cages in a temperature and humidity-controlled room on a 12 h/12 h light/dark cycle. Mice were allowed to acclimatize for one week prior to undergoing any procedure and were handled 2–3 times per week. Food pellets and water were provided ad libitum and cage enrichment used additional nesting material, tunnels (cardboard and red plastic) and chew sticks. Wet food was given to mice that received intra-cranial viral vector injections in the days before and after the surgery. All procedures were carried out in compliance with the project license and in alignment with the Animals (Scientific Procedures) Act 1986 by Home Office Personal Licensees.

### Primary cortical culture and multielectrode array measurements

Brains of C57BL/6J mice were extracted on postnatal day 0 in wash buffer consisting of HBBS (Sigma-Aldrich, H9394), 5 mM HEPES (Invitrogen, 15630056) and 0.2% Penicillin-Streptomycin solution (ThermoFisher, 15070063). Cortices were resected, and cut into smaller pieces in wash buffer supplemented with 20% FBS (dissection buffer). Cortical pieces were then washed with wash buffer to remove residual FBS and incubated in digestion buffer (137 mM NaCl, 25 mM HEPES, 7 mM Na_2_HPO_4_, 5 mM KCl, pH 7.2), which had been supplemented with 0.3% trypsin (Thermo, 15090046) and DNase (Sigma-Aldrich, D5024-150KU) for 10 min at 37 °C. Dissection buffer was used to neutralize the digestion and the tissue was washed with wash buffer. The cortical pieces were mechanically dissociated in wash buffer supplemented with 12 mM MgSO_4_ and DNase. The resulting cell solution was passed through a strainer (Fisherbrand, 11873402), spun down at 1000 RPM for 5 min and the cell pellet resuspended in neurobasal A medium (ThermoFisher, 10888022) supplemented with 2% B27 (ThermoFisher, 17504044), 0.5 mM GlutaMAX (ThermoFisher, 35050061) and 0.2% Penicillin-Streptomycin solution (NBA^++^ media). An aliquot of cells was mixed with trypan blue and added onto a hemocytometer to count viable cells.

Forty-two thousand cells were plated in a 5 µL drop onto each electrode field of a 24-well Cytoview MEA plate (Axion BioSystems, M384-tMEA-24W), which had been coated with poly-L-lysine (Sigma-Aldrich, P4707) and laminin (Sigma-Aldrich, L2020) at a ratio of 32:1 to promote cell adhesion, and allowed to settle down at 37 °C, 5% CO_2_ for 1–2 h with sterile water in the reservoirs surrounding the wells. 500 μL warm media was added to each well subsequently. MEA plates were maintained at 37 °C, 5% CO_2_, with sterile water changes occurring each week, and media changes occurring on DIV2, DIV7, DIV12 and DIV18 (after recordings where applicable). AAVs were diluted in NBA^++^ media to a multiplicity of infection (MOI) of 80,000, consistent with commonly used in vitro doses,^91–95^ and then added to each well on DIV6.

MEA activity was recorded (37 °C, 5% CO_2_) following a 5-min acclimatization. AxIS Navigator (v3.7.1) was used to estimate viability using cell impedance and to measure spontaneous electrical activity at a sampling rate of 12,500 Hz for 10 min. Spikes were detected using a threshold of six times the standard deviation of the baseline, and bursts were defined as five or more consecutive spikes with a maximum inter-spike interval of 50 ms. Network bursts were defined as bursts detected in at least 67% of electrodes.

DPH was dissolved in DMSO to a molarity of 20 mM and then serially diluted in DMSO and NBA^++^, and added to each well at a final concentration of 200 nM with a final DMSO content of 0.01%. Following a 24 h-incubation period, the drug-containing medium was replaced with fresh/conditioned medium (50/50) and an additional datapoint collected the day after (DIV22) to measure the recovery from DPH exposure.

### Viral vector injections

C57BL/6J mice aged 8–9 weeks were anesthetized with isoflurane (5% induction; 2% maintenance) and injected with buprenorphine (0.15 mg/kg) and Metacam (1.5 mg/kg) subcutaneously for post-operative analgesia, followed by a local injection of subcutaneous bupivacaine on the scalp (0.01 mL of 1.25 mg/mL stock as an incisional block). Mice received intra-hippocampal injections of AAV9 particles at 100 nL/min using a microinjection pump (WPI, Ltd) and Hamilton syringe (900 series, 5 μl, 34-gauge flat-end needle, EssLab Ltd). Mice prepared for brain slice electrophysiology experiments received 500 nL of a mixture containing AAV9-CaMKII-HA-GRANPA (5 × 10^12^ vg/mL) + AAV9-CaMKII-GFP (1 × 10^12^ vg/mL) diluted in sterile saline bilaterally into the hippocampus using the coordinates AP = −3.1 mm, ML = ± 3.3 mm, DV = −3.5 and −2.5 mm from pia (250 nL injection at each depth). Mice prepared for OFT and PTZ experiments received 1.5 μL of AAV9-CaMKII-HA-GRANPA (5 × 10^12^ vg/mL) or AAV9-CaMKII-dscGFP (5 × 10^12^ vg/mL) diluted in sterile saline bilaterally into the hippocampus using the coordinates AP = −3 mm ML = ± 3 mm DV = −3.5, −3 & −2.5 mm from pia (500 nL injection at each depth). The skin was closed with simple interrupted sutures, and mice were allowed to recover in a heat box at 37 °C until awake and active. Following surgery mice were given subcutaneous saline (0.5 mL) to aid recovery.

### Brain slice electrophysiology

Three weeks following viral vector injections mice were anesthetized (5% isoflurane) and given a lethal dose of sodium pentobarbital (600 mg/kg, intraperitoneally). Once complete loss of reflexes was ascertained, mice were transcardially perfused with ice-cold, oxygenated (95% O_2_/5% CO_2_) artificial CSF (aCSF) containing (in mM): NaCl (87), KCl (2.5), NaHCO_3_ (25), NaH_2_PO_4_ (1.25), glucose (25), sucrose (75), MgCl_2_ (7), CaCl_2_ (0.5). The brain was then removed, placed into a dissecting dish, and surrounded by ice-cold aCSF. The brain was placed onto its ventral surface and the cerebellum and brainstem removed from the forebrain. A longitudinal cut along the midline separated the two hemispheres of the forebrain which were then placed onto the freshly cut medial surface. A small section of dorsal cortical surface (10% of brain volume, 10° acute to the sagittal plane) was removed^96^ and the freshly cut dorsal surface was glued to the vibratome stage using a small amount of cyanoacrylate glue. The stage was placed into the slicing chamber surrounded with iced, slicing aCSF which was continually oxygenated with 95% O_2_/5% CO_2_. 400 µm horizontal brain slices were collected at a slicing speed of 0.08 mm/s and placed into a recovery chamber containing slicing aCSF heated to 35 °C for 30 min. Following recovery, the chamber was removed from the water bath and allowed to cool to RT. The slices were stored at RT until required.

Individual slices were transferred to the recording chamber and continuously perfused with oxygenated recording aCSF warmed to 35 °C, containing (in mM): NaCl (125), KCl (2.5), Glucose (25), NaHCO_3_ (25), NaH_2_PO_4_ (1.25), MgCl_2_ (2), CaCl_2_ (1). Slices were left for 5 min in the recording chamber to stabilise before recordings were carried out. Slices were first checked for GFP immunofluorescence (455 nm LED, Thorlabs and GFP filter set, Chroma) in the CA3 *stratum pyramidale* region and the CA1 *stratum radiatum* region, indicating viral vector targeting to the Schaffer collaterals. Only slices that contained GFP in both regions were used for experiments. A tungsten, concentric bipolar stimulating electrode (WPI, TM33CCINS) and a glass recording electrode (borosilicate glass capillaries, OD 1.5 mm, ID 0.86 mm) were placed in the *stratum radiatum* of CA1, at least 300 µm apart. Glass recording electrodes were filled with the recording aCSF as described above. Field excitatory post synaptic potentials (fEPSPs) were elicited using electrical stimulation (Digitimer DS3; 100 µs pulses) of the Schaffer collaterals every 30 s at increasing current amplitudes (20–200 µA) to produce an input-output curve. The current required to elicit a response that was 70% of the maximum amplitude was used for experiments.

Field recordings were made with a Multiclamp 700B amplifier (Molecular Devices), filtered online at 6 kHz and digitised at 50 kHz (NI-USB6341) using winWCP software (John Dempster, University of Strathclyde, Glasgow, UK). Slices were perfused with oxygenated recording solution at a rate of 3 mL/min. fEPSPs were elicited every 30 s during a 15-min baseline period, 15-min period in the presence of DPH (1 µM) and a 15-min washout period. Any recordings in which baseline fEPSP responses were unstable (increased or decreased by >50%) were discounted. Responses were manually measured in Clampfit (Molecular Devices; V10.7.0.3); fEPSP slopes were measured between 10-90% of the maximum amplitude on the rising phase of the response. In recordings where the fibre volley was visible, the absolute maximum amplitude was measured as a control for the duration of the experiment.

### Behavioural testing

DPH was dissolved in DMSO to make a 0.5 M stock solution and then subsequently dissolved in saline to a final concentration of 0.1 mg/mL (0.07% DMSO). For the vehicle solution, DMSO was dissolved in saline to a final concentration of 0.07%. Mice were acclimatized to the experimental room for 20–30 min prior to behavioural testing. Mice received 1 mg/kg DPH or vehicle by I.P. injection and were then placed in a holding cage. Following a 10-min wait, tunnel-handled mice were placed in a 30x30 cm white arena under dim light for 10 min, and the exploration was recorded with a camera. Thigmotaxis was defined as % time spent outside the 6x6 cm central area. Upon trial completion, each mouse was placed in a holding cage to avoid disturbing the other mice in the homecage yet to be trialled. In-between trials, the open field arena was cleaned with 70% ethanol to remove residual odours. Animal movements were automatically detected using ANY-maze software (v7.52) and analysed by an experimenter blind to viral vector identity. Fecal boli were counted at the end of each test, and unsupported rearing events were counted on video.

### Acute seizure model

Mice received an I.P. injection of 1 mg/kg DPH (dissolved in saline, 0.07% DMSO), followed by an I.P. injection of 50 mg/kg PTZ (dissolved in saline) 10 min later. The onset and progression of acute seizures were monitored for 30 min following chemoconvulsant administration, and scored using a modified Racine scale.^97^ At the end of the study, mice were sacrificed by trans-cardiac perfusion and their brains preserved for subsequent staining and/or imaging using 4% paraformaldehyde (exchanged after 24 h with PBS).

### Immunohistochemistry

Fixed brains were sliced into 30 μm coronal sections using a vibrating microtome (VT1000S, Leica). Brain slices were permeabilized in PBS containing 0.3% Triton X-100 (PBST; Sigma-Aldrich, T9284) for 30 min, blocked in 0.3% PBST with 8% NGS (Sigma-Aldrich, G9023) for 1 h and incubated in 0.2% PBST with 4% NGS and 1:1000 dilution of anti-HA.11 antibody (BioLegend, 901501) overnight at 4 °C. After four PBS washes, slices were incubated in PBS with 2% NGS and 1:1000 dilution of an anti-mouse 594 secondary antibody (Invitrogen, A-11005) for 2 h. Following another four PBS washes, slices were mounted onto microscope slides (Polysciences, 26414-1) using mounting medium (Abcam, ab104139). Tissue was imaged with an Axio Imager A1 fluorescence microscope using AxioVision software (Zeiss).

### Chronic epilepsy model

Mice were anaesthetized with isoflurane (5% induction; 1–2% maintenance), and their heads were shaved and disinfected with iodine. They were then positioned in a stereotaxic frame and buprenorphine (0.15 mg/kg) and meloxicam (1.5 mg/kg) were administered subcutaneously for perioperative analgesia. A midline incision was made to expose the skull, which was cleared of connective tissue. Using a robotic stereotaxic system (Neurostar), a small burr hole (~0.5 mm diameter) was drilled over the dorsal hippocampus (coordinates: AP = –2.0 mm, ML = 1.5 mm, DV = –2 mm from bregma). A total volume of 50 nL kainic acid (0.2 µg/µL) was injected at a rate of 100 nL/min using a microinjection pump (Neurostar) and a Hamilton syringe (900 series, 5 μL, 34-gauge flat-end needle, EssLab Ltd). The needle was left in place for 1 min before being withdrawn. The scalp was sutured, and animals were placed in a heated recovery chamber for 10 min, moved into a new cage and monitored for 40 min to assess the occurrence of convulsions.

Two weeks later, mice were re-anesthetized using the same protocol. Anesthetic depth was confirmed by the absence of hind paw withdrawal reflex and monitored continuously. The head and neck were shaved, and the eyes protected with Viscotears. Lidocaine and iodine were applied to the scalp. Buprenorphine (0.15 mg/kg), meloxicam (1.5 mg/kg), saline (0.5 mL, without glucose), and Betamox (0.1 mL) were administered subcutaneously. Injections of 500 nL of AAV9-CaMKII-GRANPA (5 × 10¹² vg/mL) were made into both the dorsal and ventral hippocampi bilaterally (coordinates: dorsal, AP = –2.0 mm, ML = ± 1.5 mm, DV = –2.2 mm; ventral, AP = –3.0 mm, ML = ± 3.0 mm, DV = –3.5 mm from bregma) using a Hamilton syringe. An electrode made of 140 µm Teflon-coated platinum–iridium wire was implanted at the site of previous kainate injection (coordinates: AP = –2.0 mm, ML = 1.5 mm, DV = –2.1 mm from bregma). A wireless telemetry transmitter (model A3048P2, Open Source Instruments) was inserted through the same cranial incision, guided subcutaneously through the neck, and placed on the lower left flank. Recording wires were secured to the skull using cyanoacrylate adhesive and dental cement to form a stable headcap. Animals were allowed to recover in a heated chamber for approximately 15 min before being returned to their home cage.

Two to three weeks later, to allow for transgene expression, EEG recordings were performed. Animals received alternating intraperitoneal injections of saline, DPH (1 mg/kg), and CLZ (1 mg/kg), with a minimum washout period of 24 h between treatments. EEG data were analysed using PyECoG software (https://github.com/KullmannLab/pyecog2). High-frequency, high-amplitude discharges were manually labelled during the 2-h period before and after each treatment, blind to the identity of the injected substance. Seizure burden (time spent in seizure state) was calculated for 120 min epochs, immediately prior to and following injection, and then normalized to the pre-injection period.

## Supplementary information

Supplementary Materials

## Data Availability

Data and reagents are available from the corresponding authors. A GRANPA plasmid has been deposited at Addgene (ID 258139). Atomic coordinates were deposited in the Protein Data Bank (PDB) under accession code 9N29. Cryo-EM maps were deposited in the Electron Microscopy Data Bank under the accession codes EMD-48827 (consensus map) and EMD-48828 (receptor focused refine map).
