# Supplementary material for: Next-generation chemogenetic inhibition using a brain-permeant non-prescription agent
Source: Signal Transduct Target Ther. 2026 Jul 3;11:259. doi: 10.1038/s41392-026-02865-4 (PMC13328383; doi:10.1038/s41392-026-02865-4)
Supplement: Supplementary file 1 — Supplementary Materials [file 41392_2026_2865_MOESM1_ESM.docx]

Supplementary Materials for

**Next-generation chemogenetic inhibition using a brain-permeant non-prescription agent**

Steven O Devenish^1^, Sahil Patel^1§^, Laura V Ussingkær^1§^, Luiz F Almeida Silva^1§^, Olivia Goff^1^, Amy Richardson^1^, Jesse I Mobbs^2,3^, Hariprasad Venugopal^4^, David M Thal^2,3^,
Dimitri M Kullmann^1^

^§^These authors contributed equally

Correspondence to:
Steven O. Devenish – s.o.devenish@gmail.com
David Thal – David.Thal@monash.edu
Dimitri M. Kullmann – d.kullmann@ucl.ac.uk

This file includes:

Supplementary Figures 1 to 26

Supplementary Tables 1 to 8


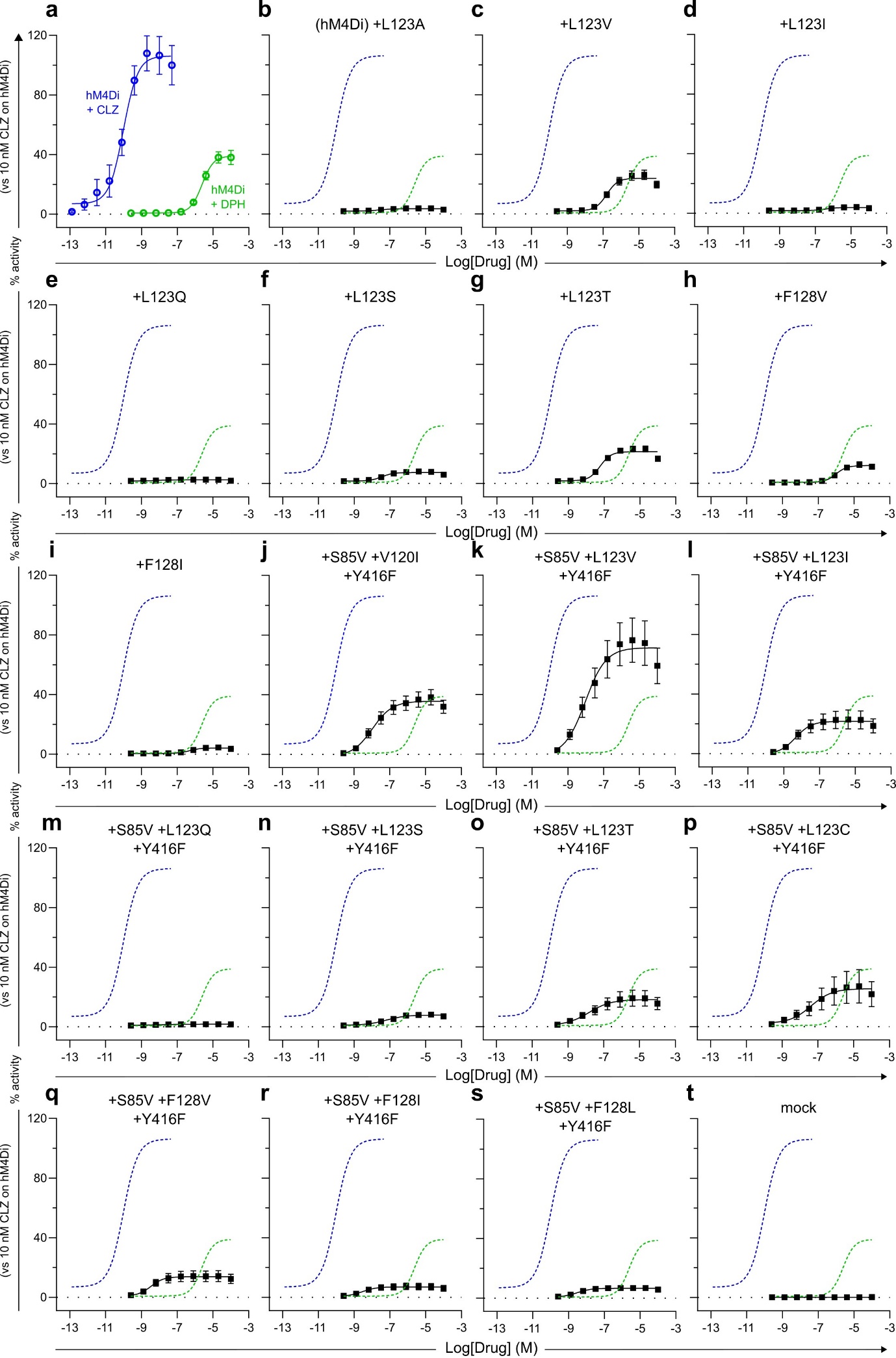


**Supplementary Figure 1. PRESTO-Tango assay for DPH acting on different mutation combinations**

**(a)** PRESTO-Tango assay measurement of the dose-response for DPH and CLZ acting on hM4Di (green and blue respectively). Data are normalized to the maximal activation of hM4Di by 10 nM CLZ. **(b – t)** Fits to the DPH and CLZ activity on hM4Di are replotted as dashed green and blue lines, respectively, with dose response data for DPH acting on the test receptor shown in black. **(t)** shows results obtained with mock-transfected cells for comparison. Data are shown as mean ± SEM.


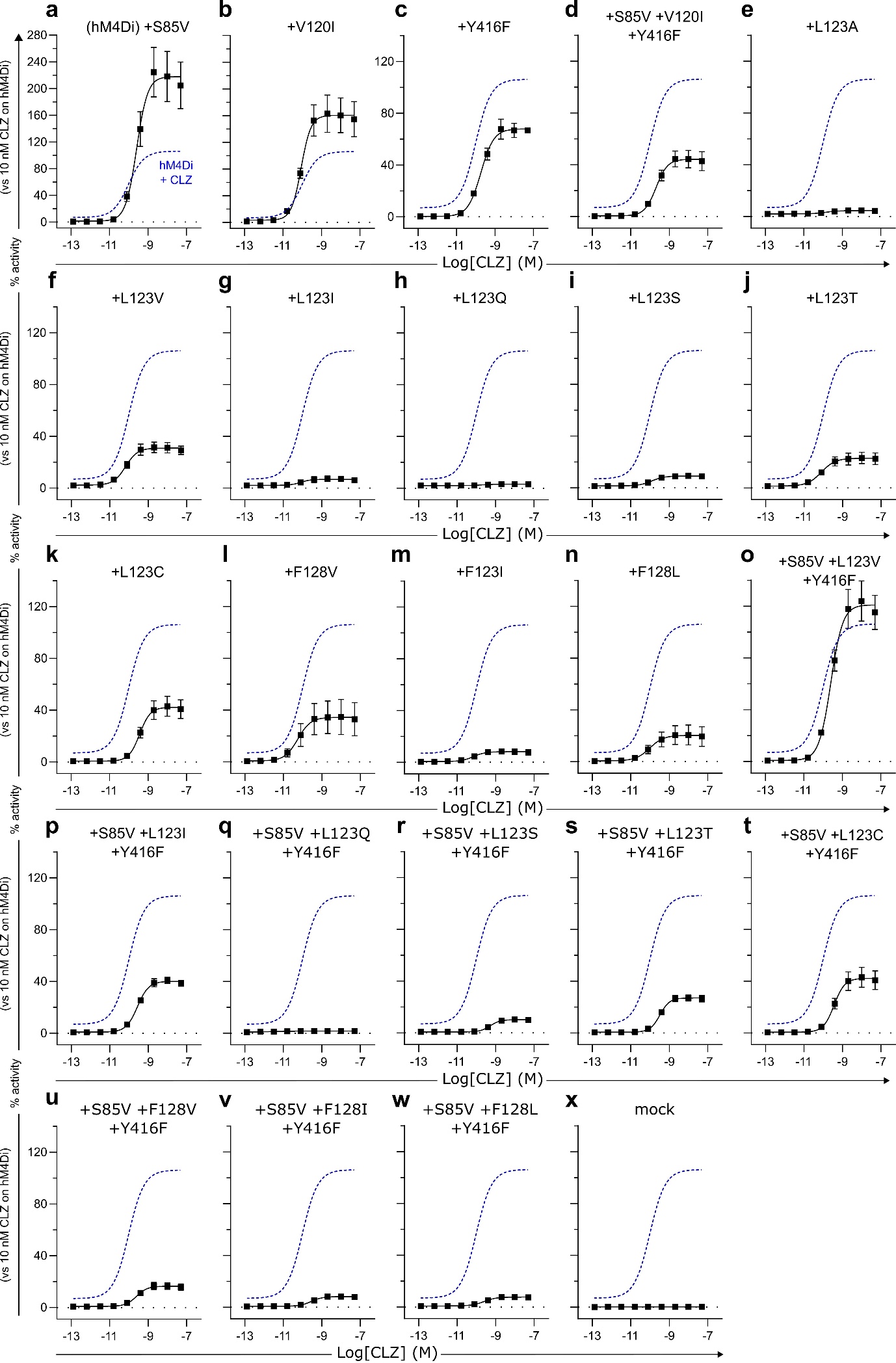


**Supplementary Figure 2. PRESTO-Tango assay for CLZ acting on different mutation combinations**

**(a – x)** PRESTO-Tango assay measurement of the response to CLZ, normalized to the maximal activation of hM4Di by 10 nM CLZ. Corresponding curve for CLZ on hM4Di is shown as dashed blue lines, dose response data for CLZ acting on the test receptor shown in black. **(x)** shows results obtained with mock-transfected cells for comparison. The Y axis maximum differs between (a, b: 280) and (c**–**x: 140). Data are shown as mean ± SEM.


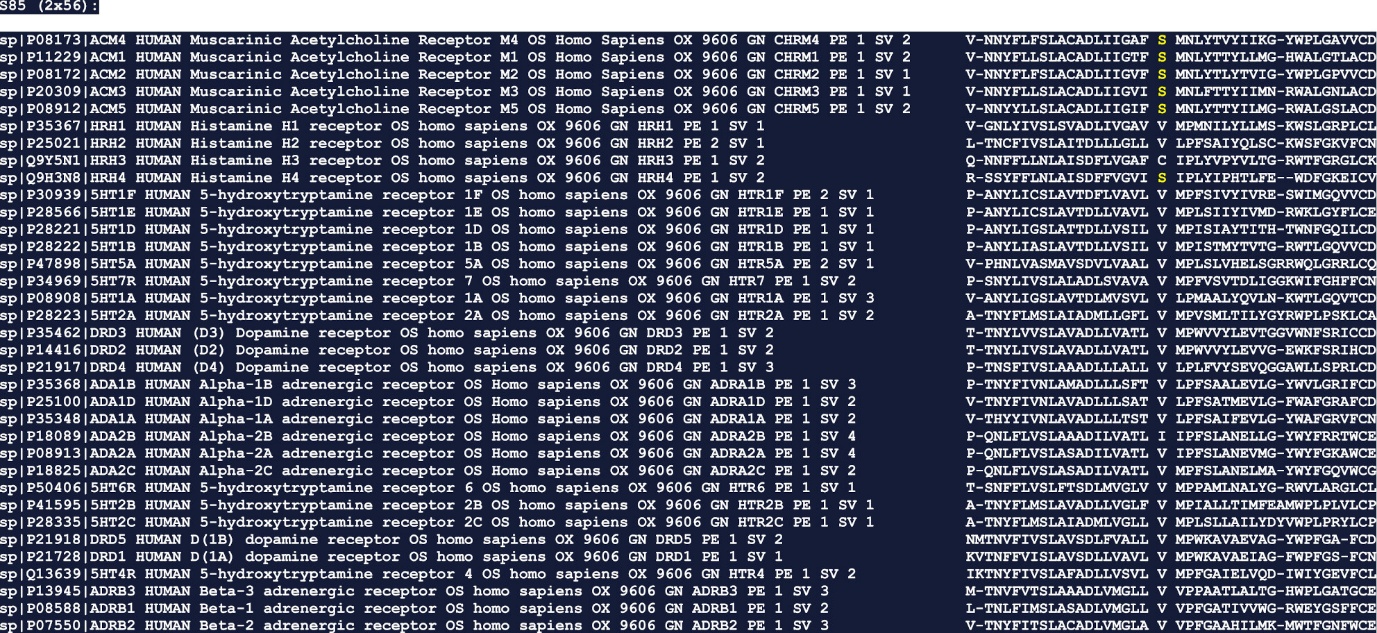


**Supplementary Figure 3. Alignment S85**

Aminergic GPCR alignment using the MUSCLE algorithm in MegaX. hM4 S85 along with all other instances of S2x56 are shown in yellow.


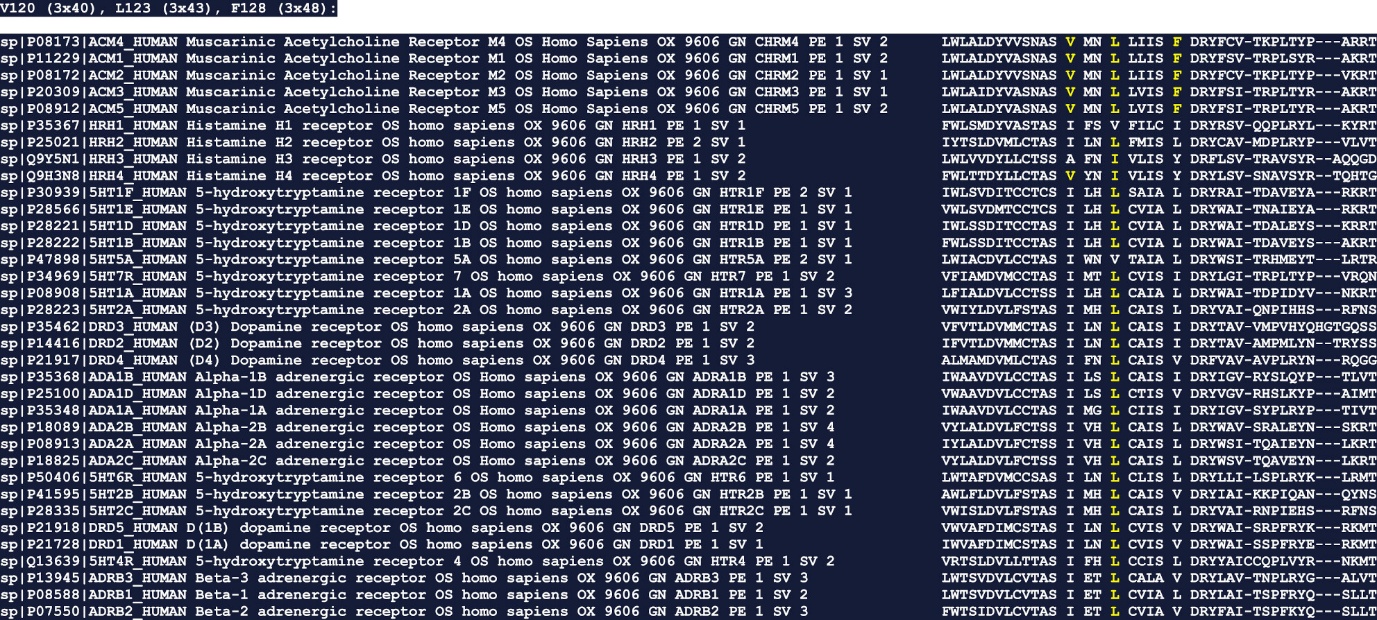


**Supplementary Figure 4. Alignment V120, L123, F128**

Aminergic GPCR alignment using the MUSCLE algorithm in MegaX. hM4 V120, L123 and F128 along with all other instances of V3x40, L3x43 and F3x48 are shown in yellow.


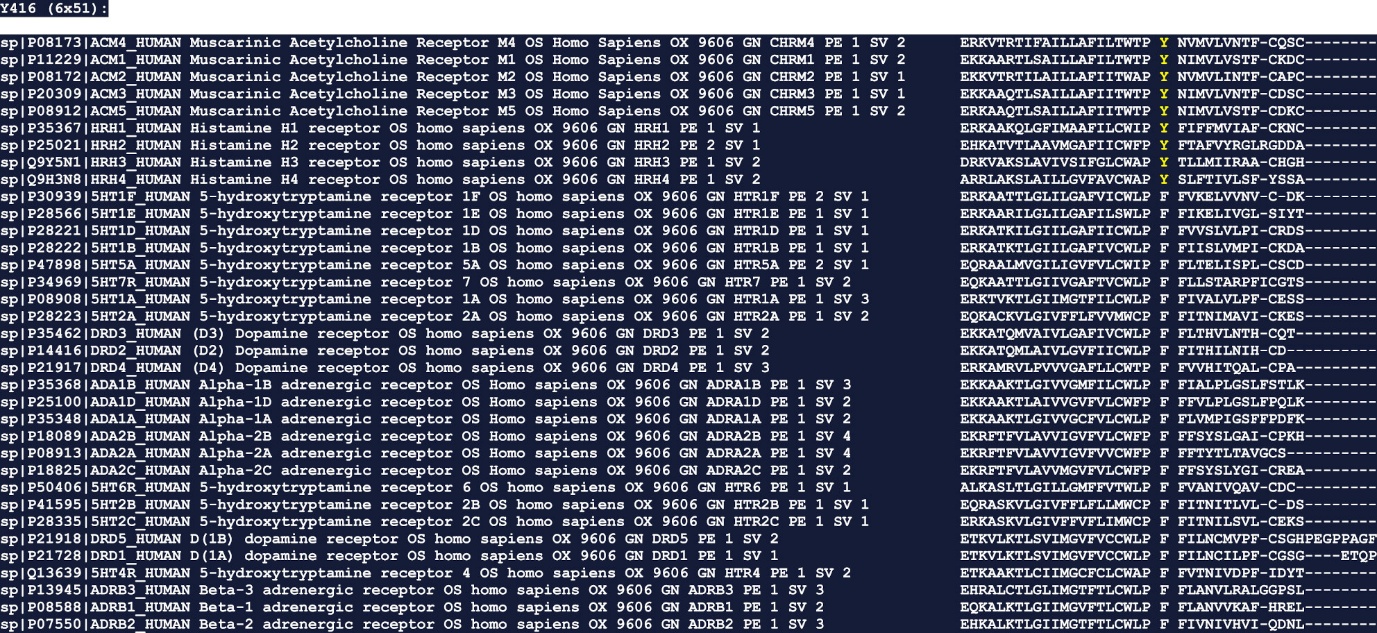


**Supplementary Figure 5. Alignment Y416**

Aminergic GPCR alignment using the MUSCLE algorithm in MegaX. hM4 Y416F along with all other instances of Y6x51 are shown in yellow.


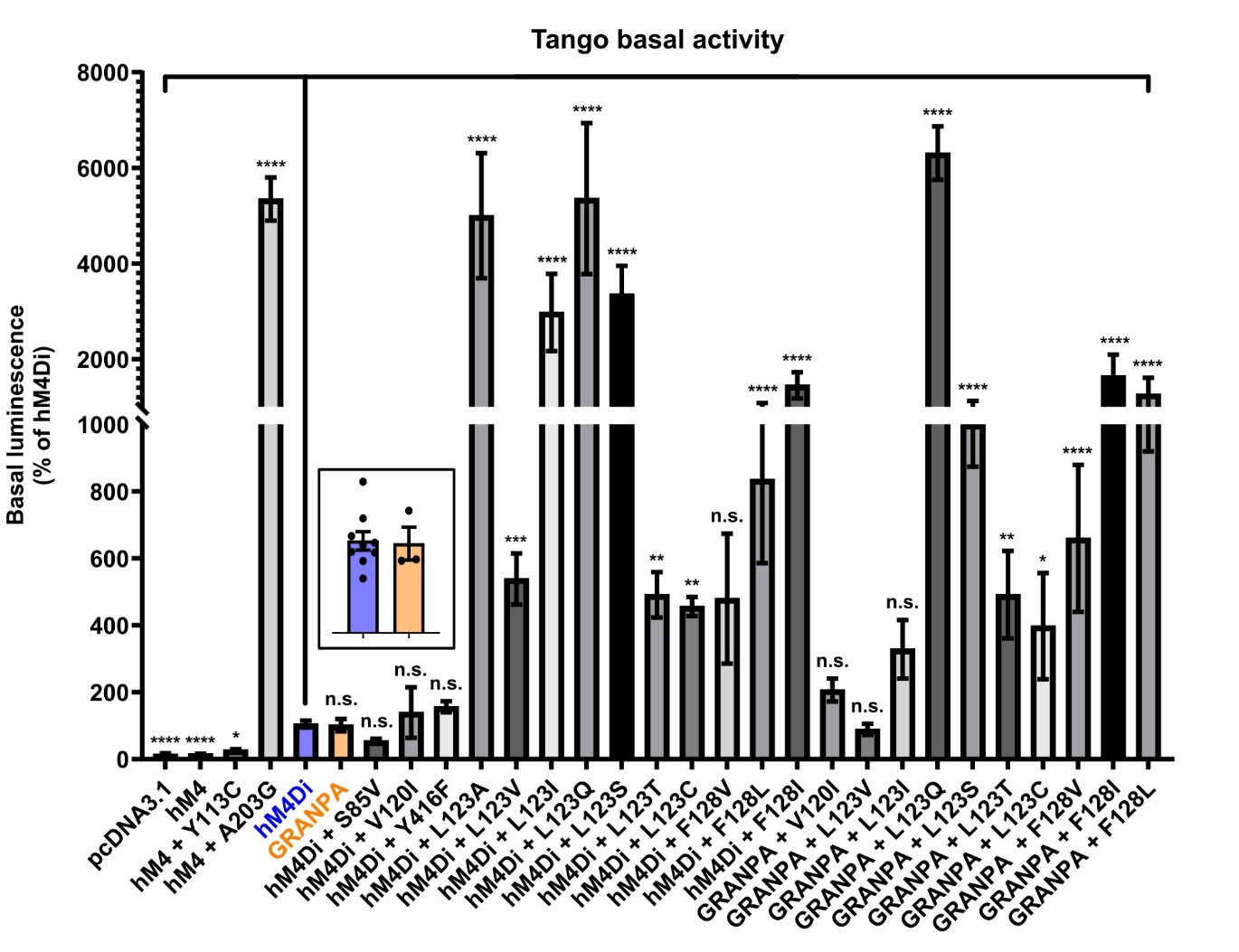


**Supplementary Figure 6. PRESTO-Tango basal activity**

PRESTO-Tango assay measurement of basal activity, normalized to the mean of hM4Di. hM4Di and GRANPA are shown in blue and orange, respectively. Insert: expansion of hM4Di and GRANPA. A one-way ANOVA with Dunnett’s test was performed, comparing hM4Di to each construct. Data are shown as mean ± SEM, n.s. p > 0.05, * p  ≤ 0.05, *** p ≤ 0.001, **** p ≤ 0.0001.


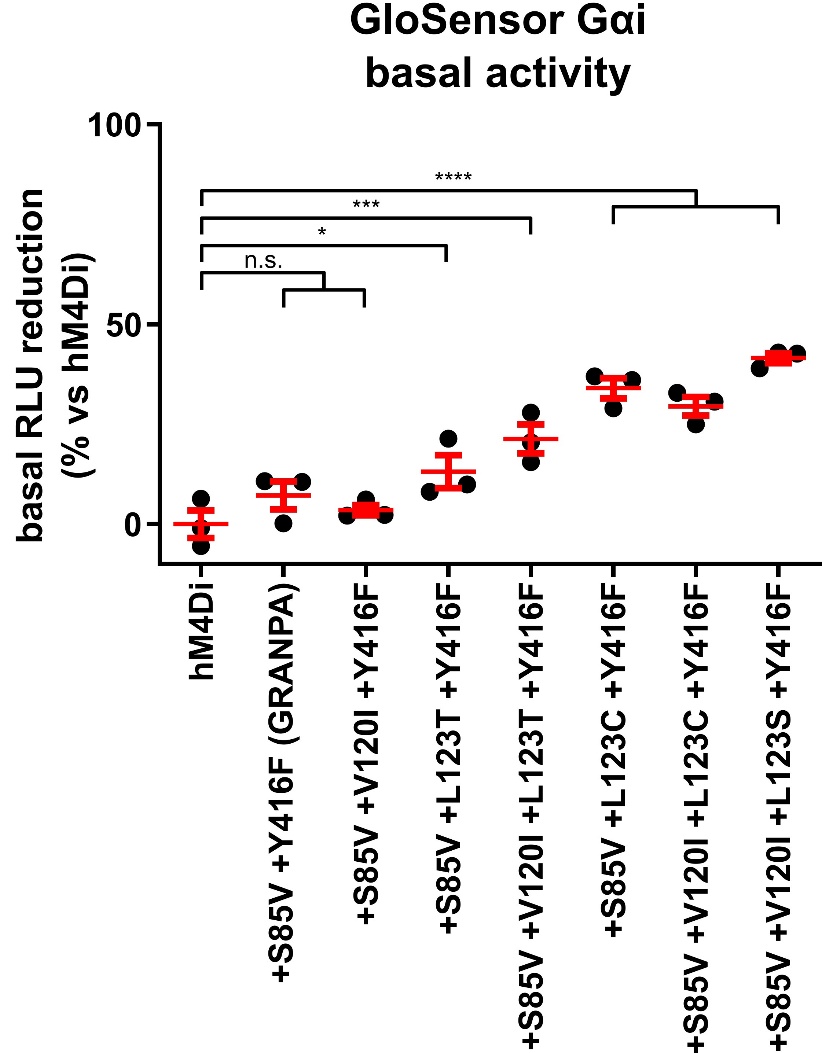


**Supplementary Figure 7. GloSensor Gαi basal activity**

GloSensor assay measurement of basal adenylyl cyclase inhibition, with native β-adrenergic receptors activated by 200 nM isoprenaline. Activity was calculated from the decrease in RLU relative to hM4Di. A larger reduction in luminescence indicates higher basal activity. A one-way ANOVA with Dunnett’s test was performed, comparing hM4Di to each construct. Data are shown as mean ± SEM, n.s. p > 0.05, * p  ≤ 0.05, *** p ≤ 0.001, **** p ≤ 0.0001.


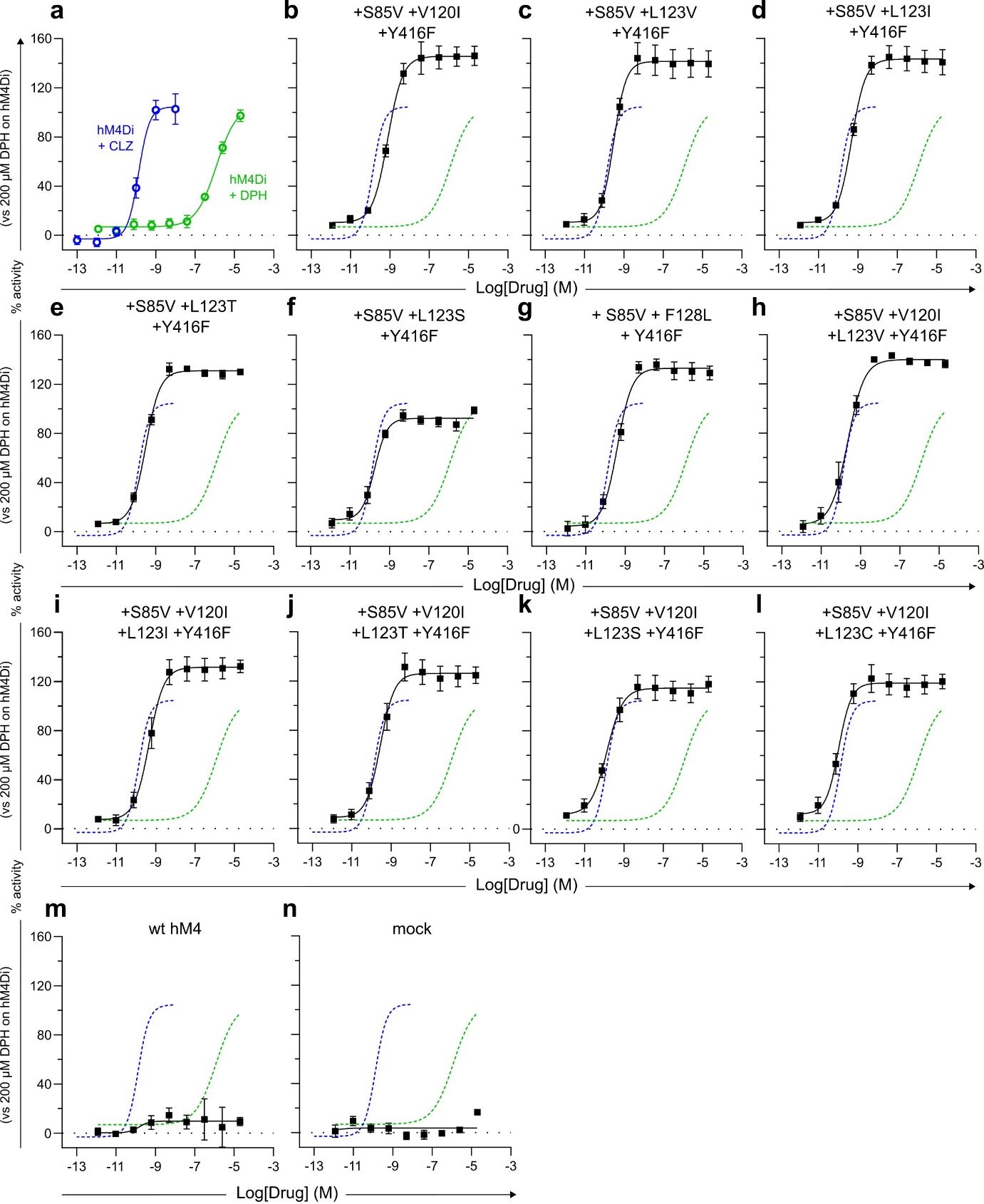


**Supplementary Figure 8. GloSensor assay for DPH-induced adenylyl cyclase inhibition with combinations of mutations**

**(a – n)** GloSensor assay measurement of adenylyl cyclase inhibition, with native β-adrenergic receptors activated by 200 nM isoprenaline. Activity was calculated from the decrease in luminescence from that obtained with isoprenaline alone, and normalized to the response of hM4Di to 200 nM DPH. Dose response data for DPH acting on the test receptor are shown in black. Dose-response data for DPH and CLZ acting on hM4Di are shown in green and blue, respectively **(a)**, and the fits (dashed green and blue curves) are replotted in panels **(b – l)**. **(m)** and **(n)** show results obtained with hM4 and mock-transfected cells for comparison. Data are shown as mean ± SEM.


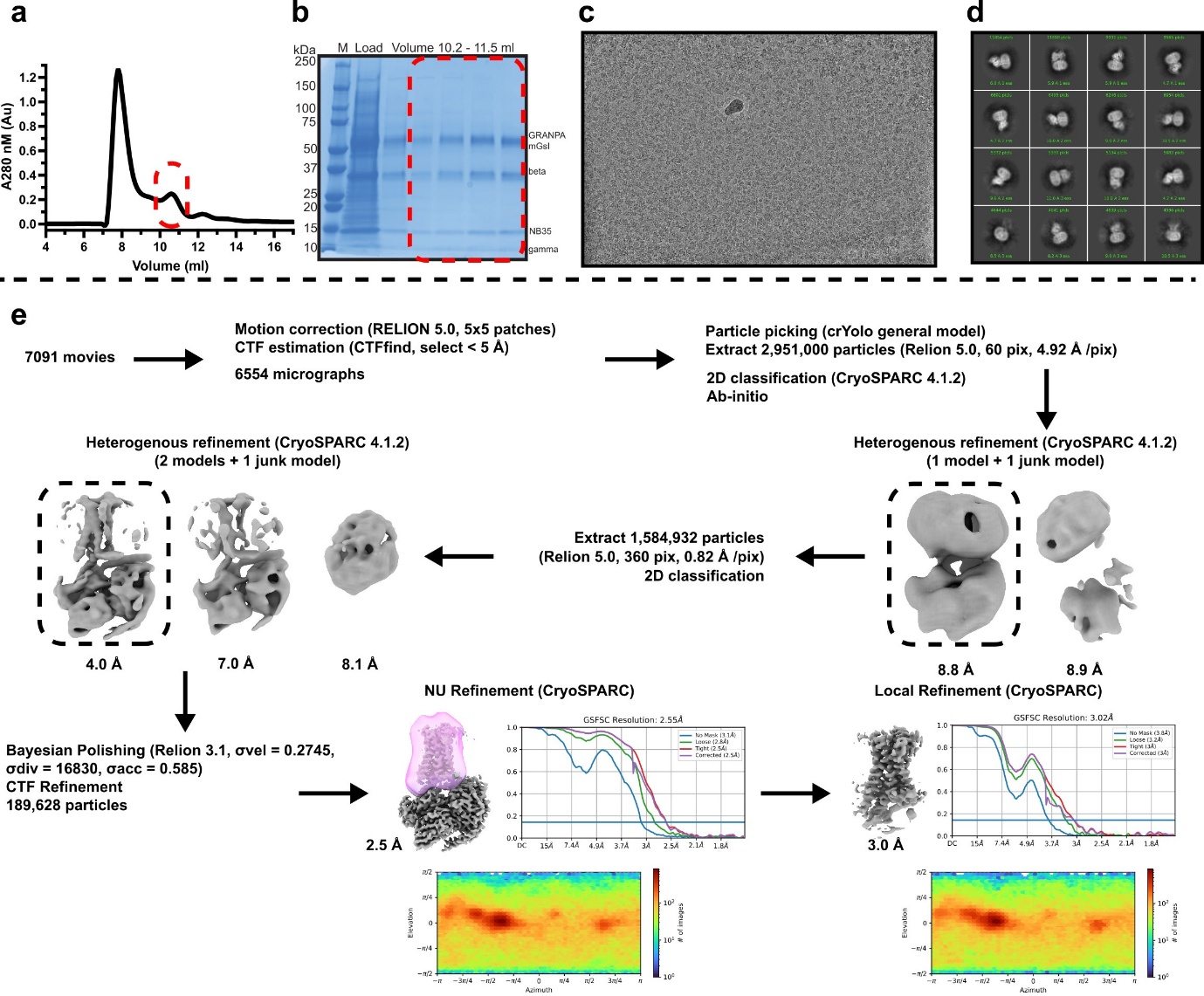


**Supplementary Figure 9. Purification and cryo-EM processing of GRANPA:DPH**

**(a)** UV absorbance chromatogram from size exclusion chromatography. The peak in the red box was pooled for cryo-EM. **(b)** Coomassie-stained SDS-PAGE of SEC fractions. M = marker. **(c)** Representative cryo-EM micrograph. **(d)** Representative 2D-classes of GRANPA:DPH from cryo-EM workflow. **(e)** cryo-EM processing workflow. Final consensus non-uniform refinement of 189K particles resulted in a 2.55 Å cryo-EM map and a local refinement of the receptor resolved to 3.0 Å.


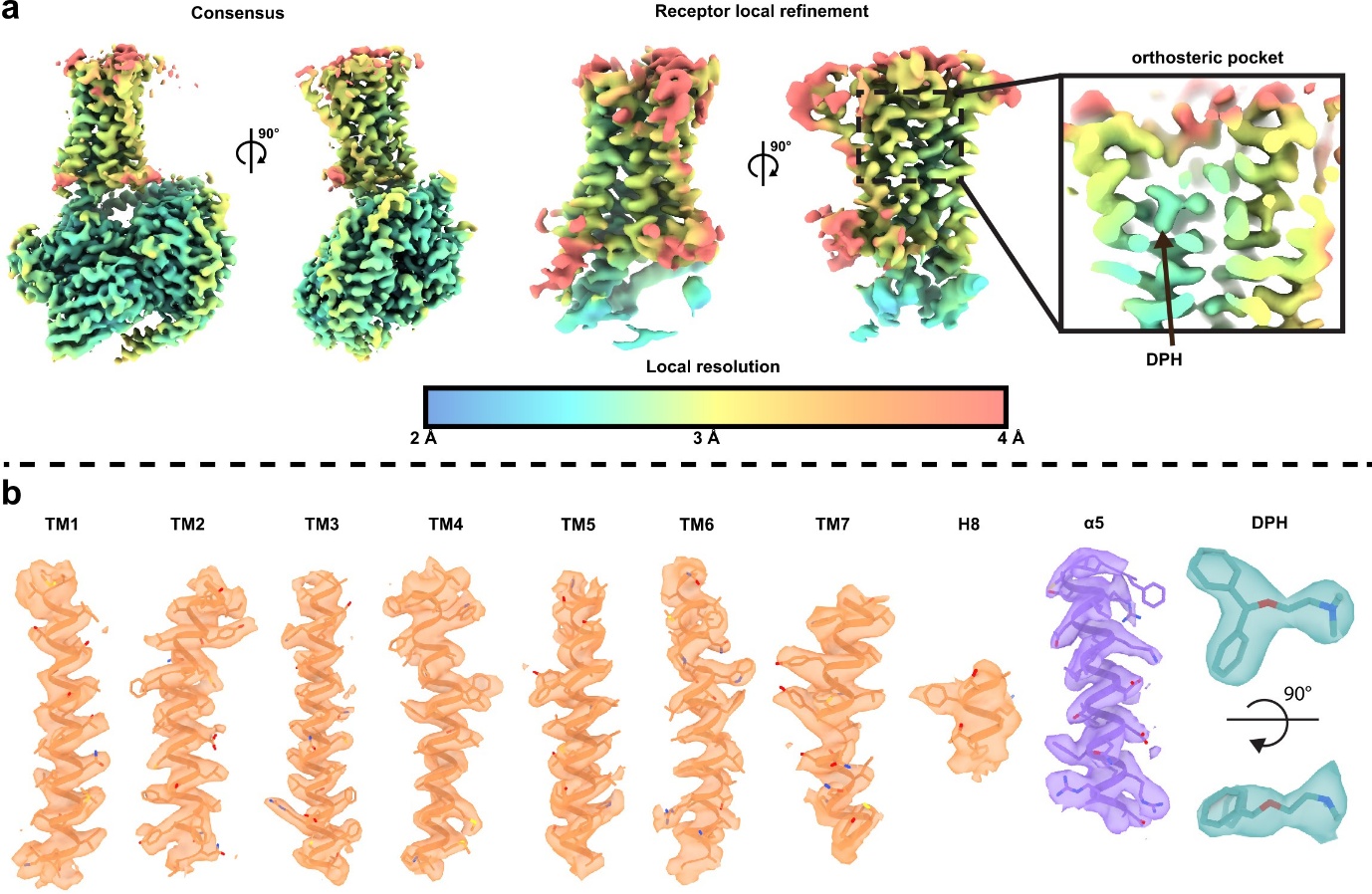


**Supplementary Figure 10 Local resolution of GRANPA:DPH cryo-EM maps**

**(a)** Local resolution estimations of consensus (left) and receptor local refinement (right) maps. Inset shows the local resolution of DPH orthosteric binding site **(b)** Cryo-EM density shown as transparent surface at a level of 0.35, overlayed on GRANPA:DPH, helices shown as cartoon and side chains as sticks.


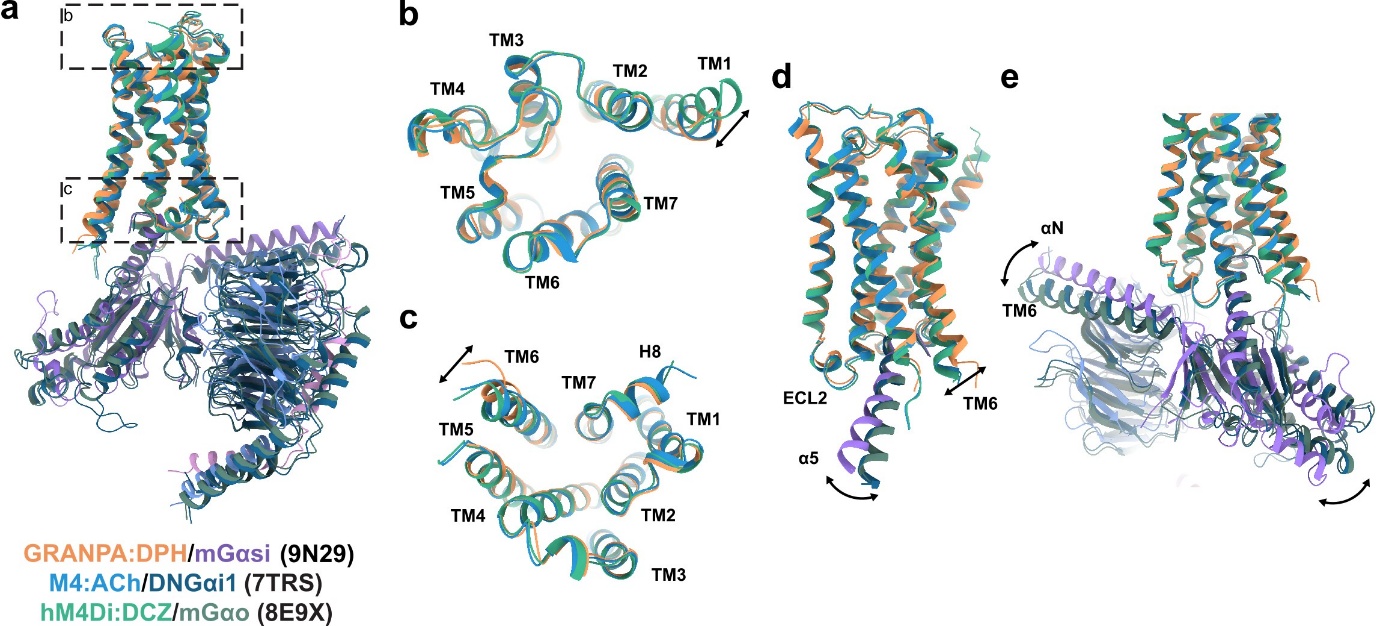


**Supplementary Figure 11. Overall structure of GRANPA:DPH**

**(a)** Structures of hM4 and ACh with DNGi1 (M4:ACh/DNGi1, PDB ID = 7TRS) and M4 DREADD and DCZ with mGo (hM4Di:DCZ/mGo, PDB ID = 8E9X) aligned to the receptor of GRANPA using Cα atoms. **(b)** Extracellular view of the receptors. **(c)** Intracellular view of the receptors. **(d)** Comparison of the α5 region and **(e)** αN region of the receptor/transducer interface.


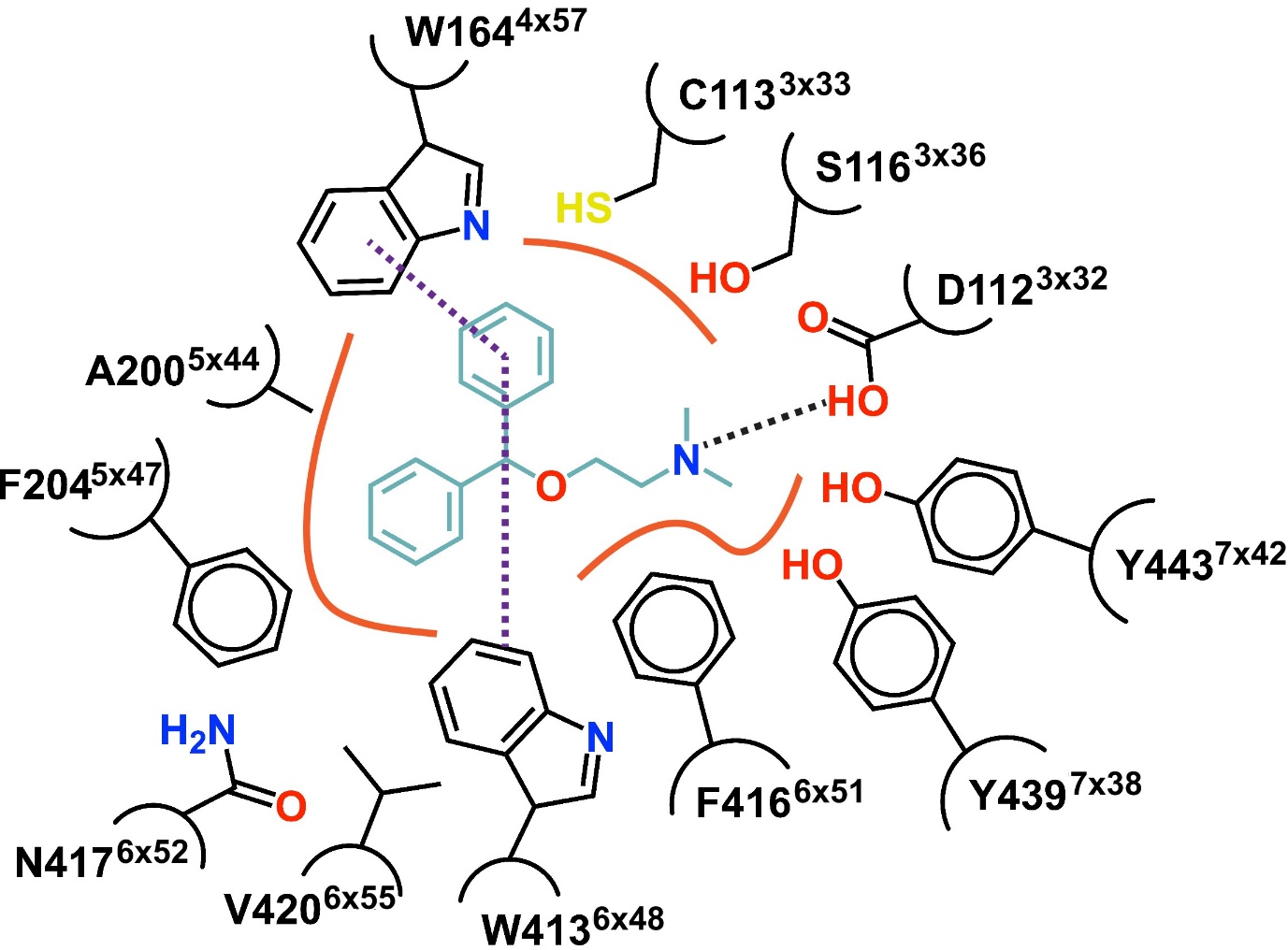


**Supplementary Figure 12. LIGPLOT**

2D representation of DPH binding to GRANPA.


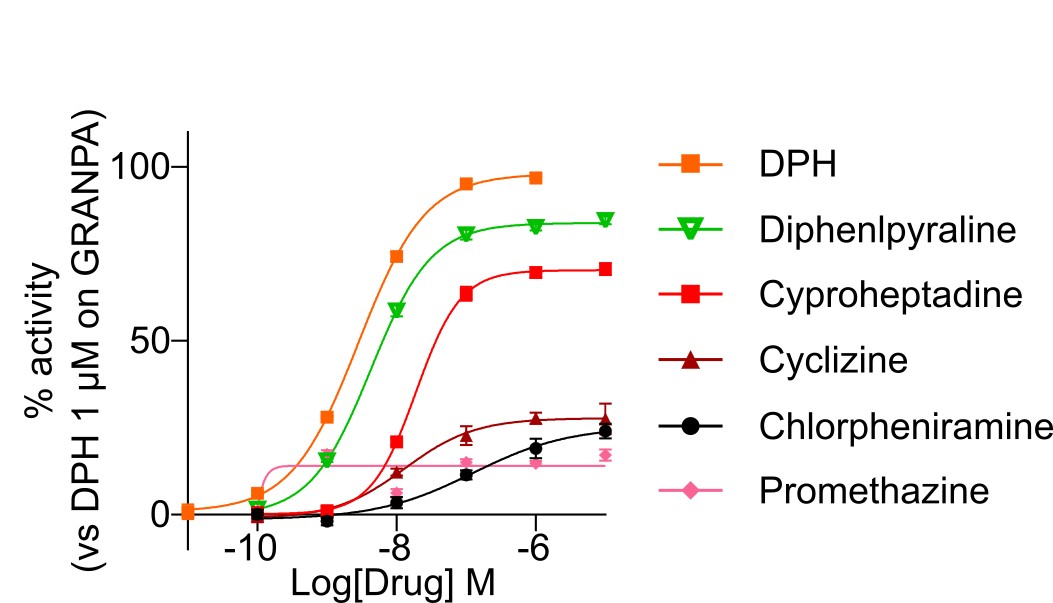


**Supplementary Figure 13. Activation of GRANPA - Gαi2 signalling by antihistamines**

TRUPATH assay measurement of GRANPA mediated Gαi2 G protein dissociation by DPH (orange), diphenylpyraline (green), cyproheptadine (red), cyclizine (brown), chlorpheniramine (black), and promethazine (pink). Activity is calculated as the reduction in raw BRET values normalized to control of GRANPA +/- DPH 1 µM. Data are shown as mean ± SEM.


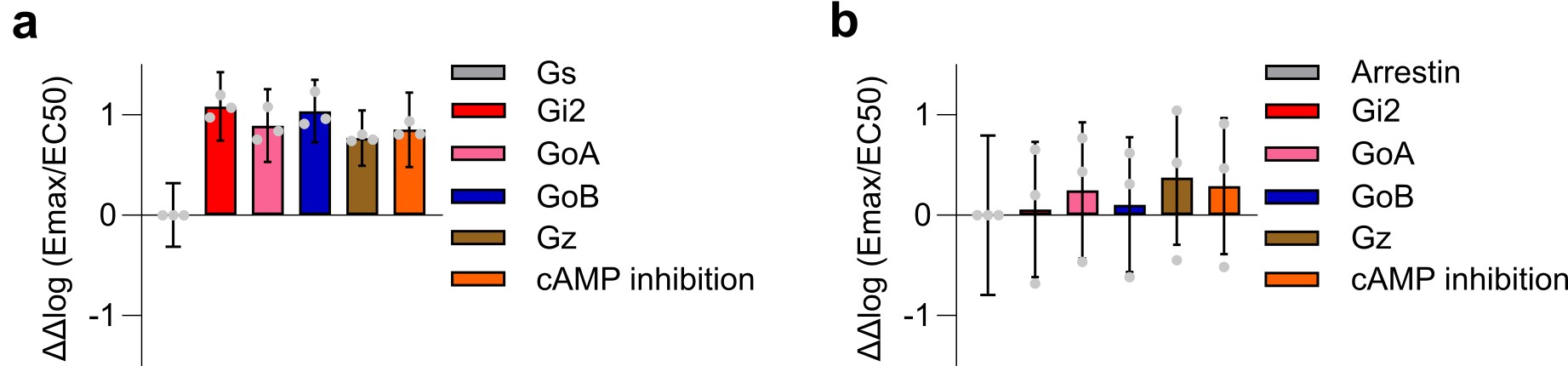


**Supplementary Figure 14.** **Bias factors for GRANPA-DPH vs GRANPA-CLZ**

**(a, b)** GRANPA-DPH bias factors (ΔΔlog (Emax/EC50)) for each pathway were calculated using hM4Di-CLZ as the relative signalling system and adenylyl cyclase stimulation (a), or β-arrestin 2 recruitment (b) as the reference pathway^1^. Data are shown as mean ± SD.


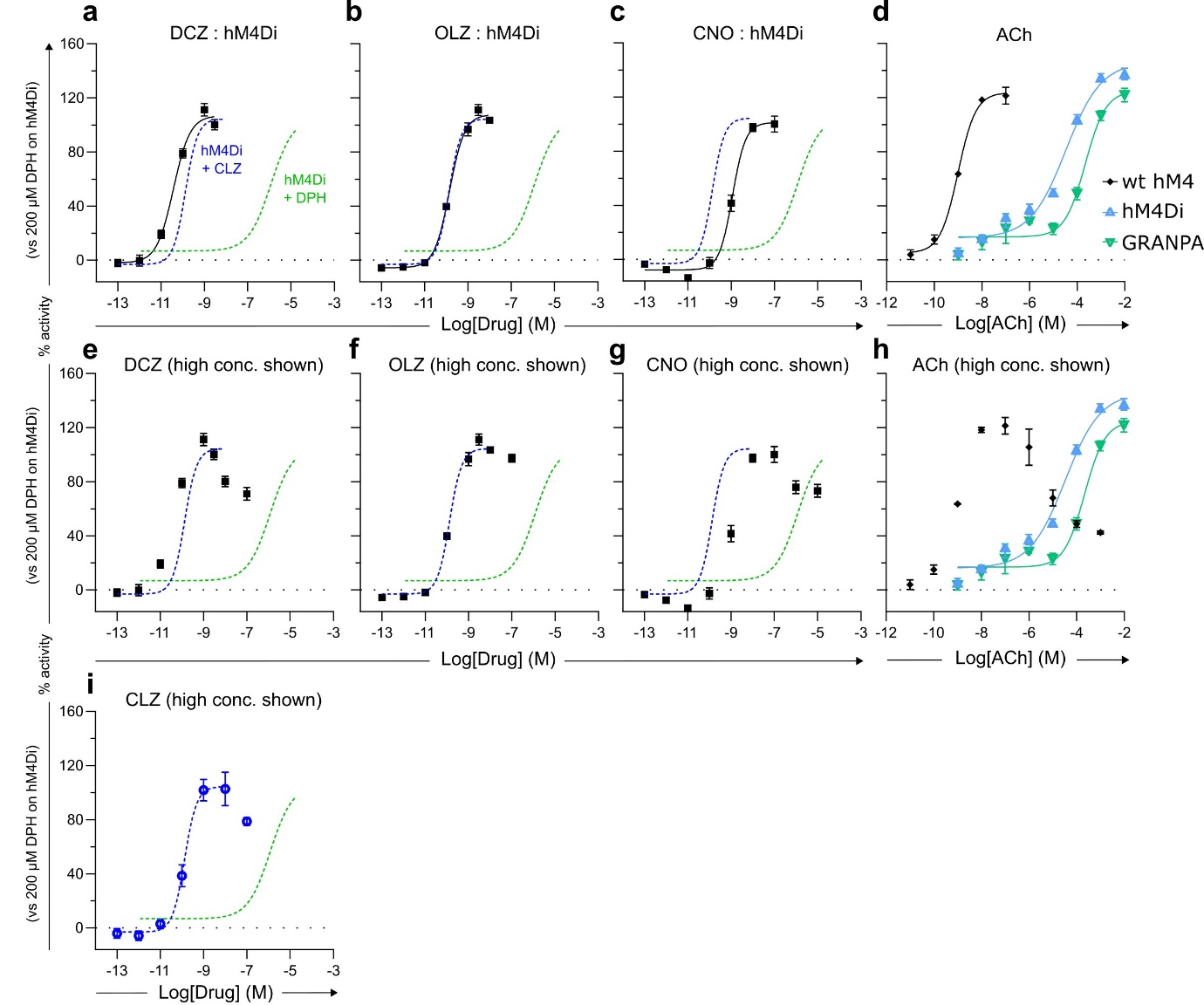


**Supplementary Figure 15. GloSensor assay for adenylyl cyclase inhibition by other ligands**

**(a – i)** GloSensor assay measurement of adenylyl cyclase inhibition, with native β-adrenergic receptors activated by 200 nM isoprenaline. Activity was calculated from the decrease in luminescence from that obtained with isoprenaline alone, and normalized to the response of hM4Di to 200 nM DPH. Corresponding curves for DPH on hM4Di and CLZ on hM4Di are shown as dashed green and blue lines, respectively, as in Supplementary Figure 8, with the drug-receptor combination indicated above shown in black. **(d)** Dose response curves for ACh acting on hM4, hM4Di, and GRANPA. **(e – i)** High concentrations of agonists result in opposing adenylyl cyclase stimulation, suppressing the Emax. When measuring inhibition of adenylyl cyclase (Gαi/o), the opposing stimulation (Gαs) by certain agonists supressed responses at higher concentrations; hence, these points were not plotted. Data are shown as mean ± SEM.


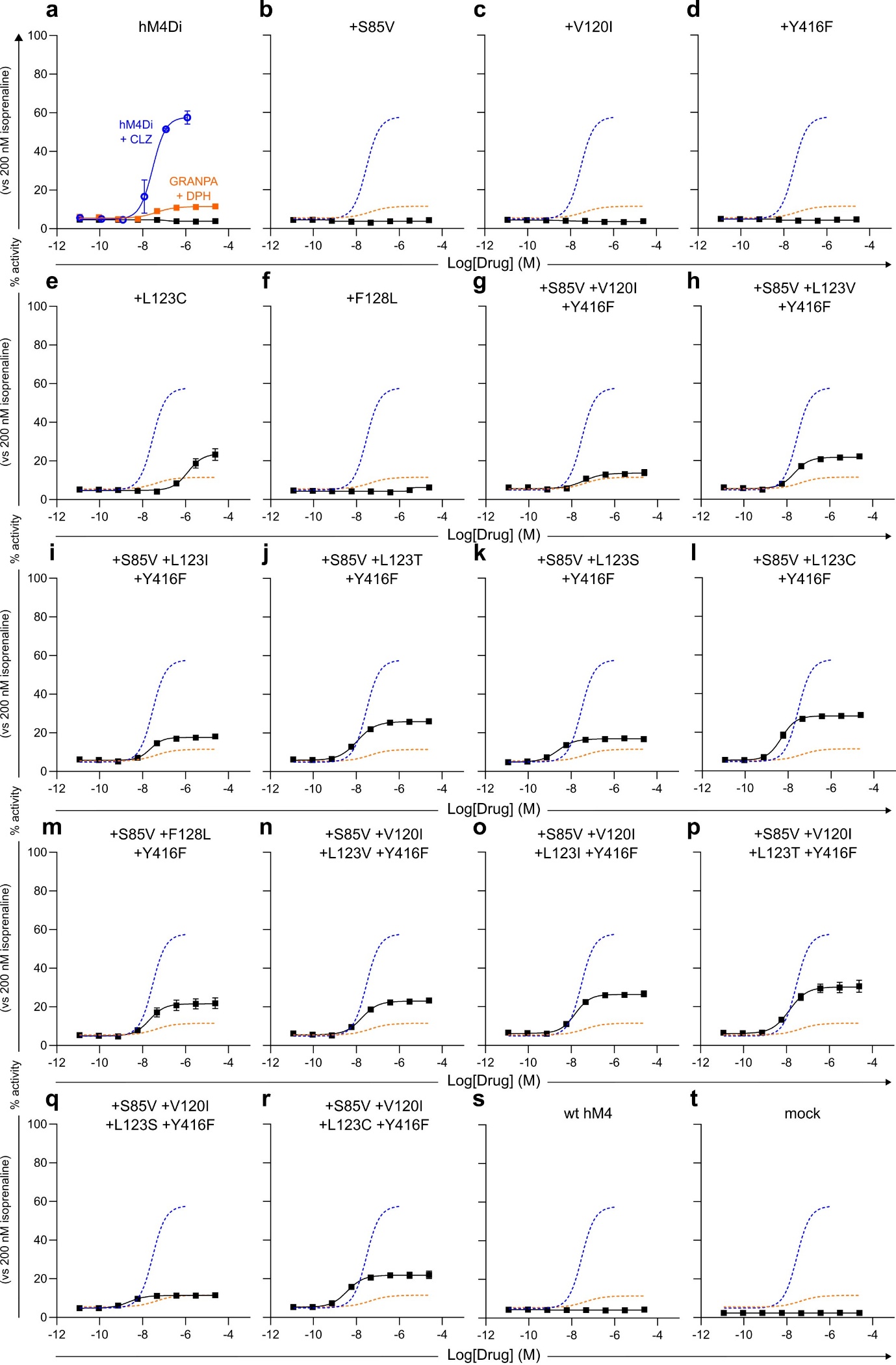


**Supplementary Figure 16. GloSensor assay for adenylyl cyclase stimulation by DPH**

**(a – t)** GloSensor assay measurement of adenylyl cyclase stimulation, normalized to the response to 200 nM isoprenaline. Corresponding curves for DPH on GRANPA and CLZ on hM4Di are shown as dashed orange and blue lines, respectively, with the effect of DPH on the receptor indicated above shown in black. **(s)** and **(t)** show results obtained with hM4 and mock-transfected cells for comparison. Data are shown as mean ± SEM.


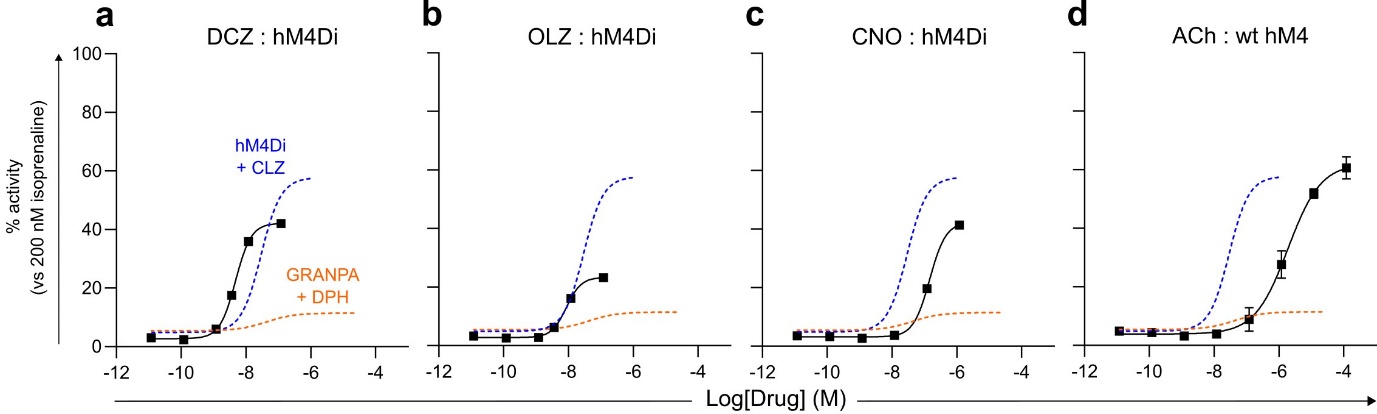


**Supplementary Figure 17. GloSensor assay for adenylyl cyclase stimulation by other ligands**

**(a–d)** GloSensor assay measurement of adenylyl cyclase stimulation, normalized to the response to 200 nM isoprenaline. Corresponding curves for DPH on GRANPA and CLZ on hM4Di are shown as dashed orange and blue lines, respectively, and overlaid on data for DCZ on hM4Di (a), OLZ on hM4Di (b), CNO on hM4Di (c), and ACh on hM4 (d). Data are shown as mean ± SEM.


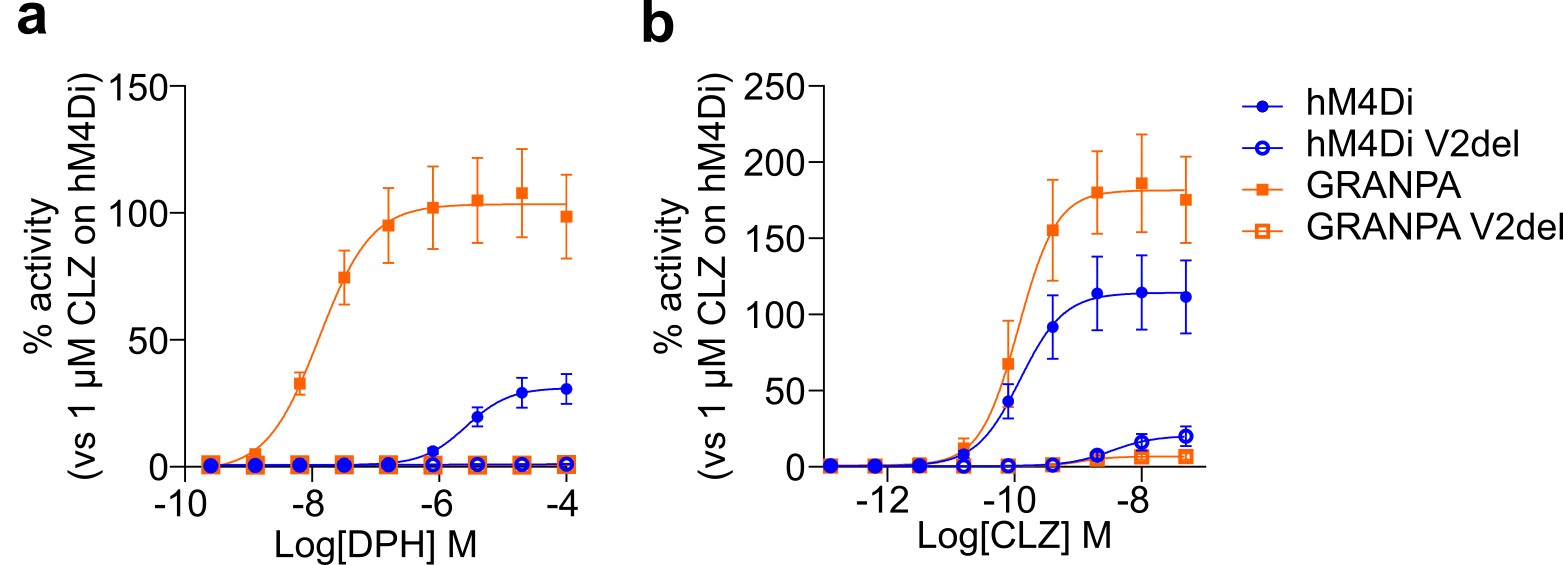


**Supplementary Figure 18. Impact of V2 tail deletion on** β-**arrestin recruitment in PRESTO-Tango assay**

**(a, b)** PRESTO-Tango assay measurement of β-arrestin recruitment by DPH (a) and CLZ (b) acting on hM4Di, hM4Di V2 tail deletion (V2del), GRANPA & GRANPA V2del. Activity is calculated as the increase of raw luminescence values normalized to control of hM4Di ± 1 µM CLZ. Data are shown as mean ± SEM.


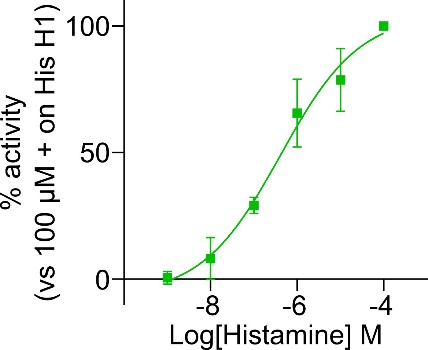


**Supplementary Figure 19. Histamine concentration-response curve**

TRUPATH assay measurement of histamine H1 mediated Gαq G protein dissociation by histamine. Activity is calculated as the reduction in raw BRET values normalized to control of histamine H1 receptor +/- histamine 100 µM. Data are shown as mean ± SEM.


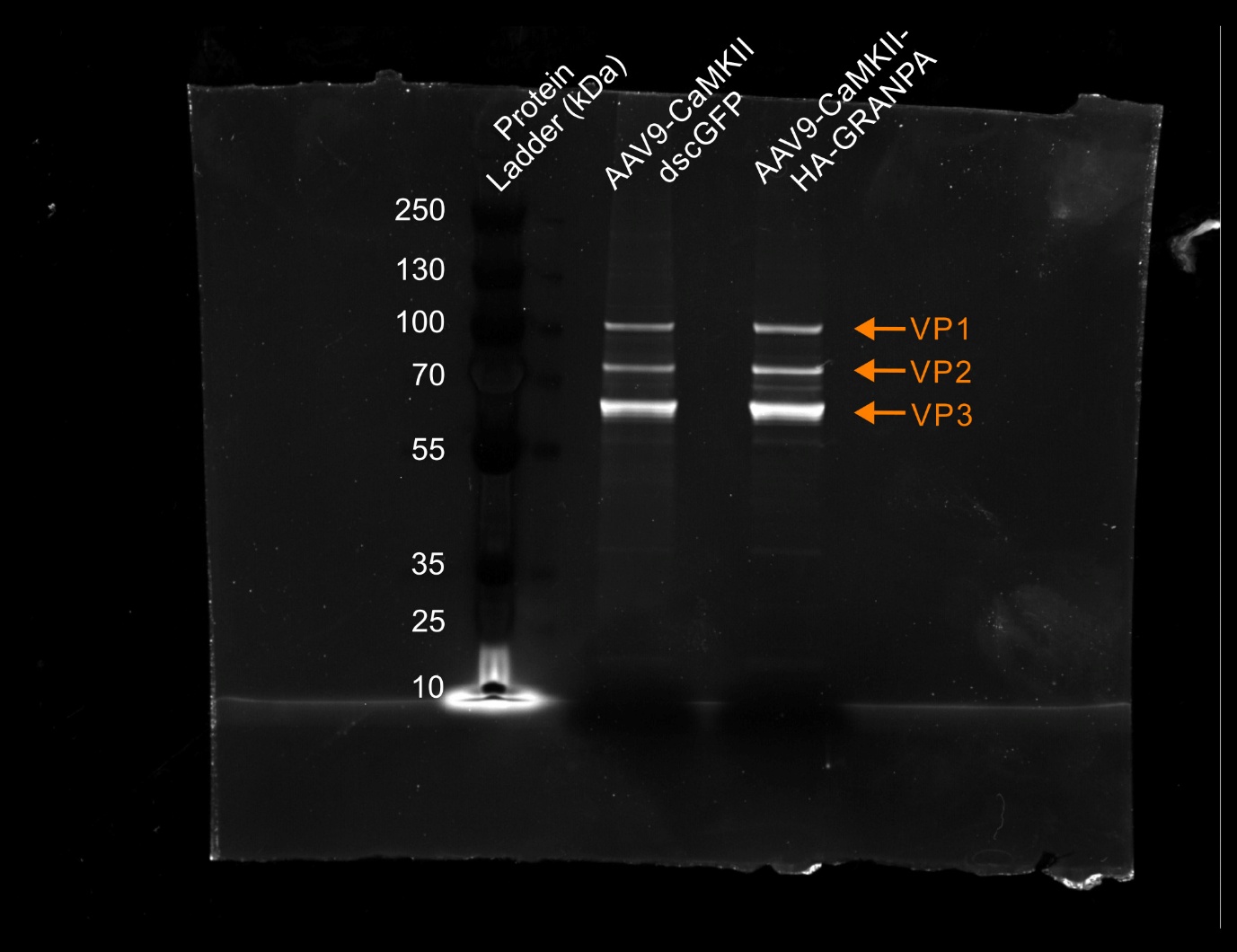


**Supplementary Figure 20. Protein purity of AAV9 preparations**

The protein content of adeno-associated viral vector serotype 9 (AAV9) preparations produced in HEK cells was determined for AAV9-CaMKII-dscGFP (lane 2) and AAV9-CaMKII-HA-GRANPA (lane 3) using SDS-PAGE. An annotated protein ladder (lane 1) indicates the presence of 3 proteins in both AAV9 preparations, which have sizes that correspond to the expected size for the AAV9 capsid proteins VP1 (~87 kDa), VP2 (~73 kDa) and VP3 (~62 kDa) at a similar ratio to the expected 1:1:10 (VP1:VP2:VP3).


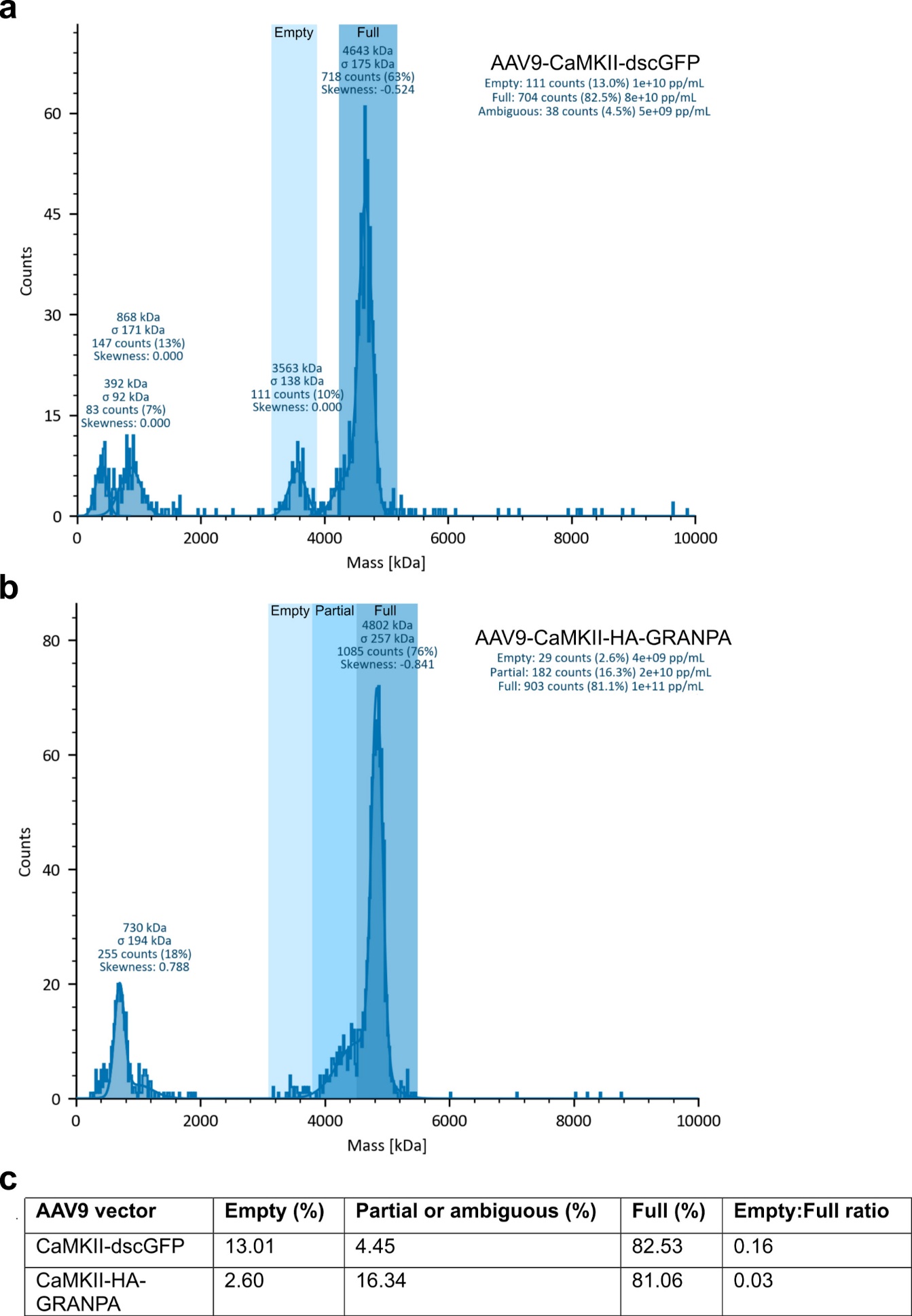


**Supplementary Figure 21. Empty/full capsid analysis of AAV9 preparations**

**(a)** Particle counts of empty and full capsids for AAV9-CaMKII-dscGFP. **(b)** Particle counts of empty and full capsids for AAV9-CaMKII-HA-GRANPA. **(c)** A summary of mass photometry findings.


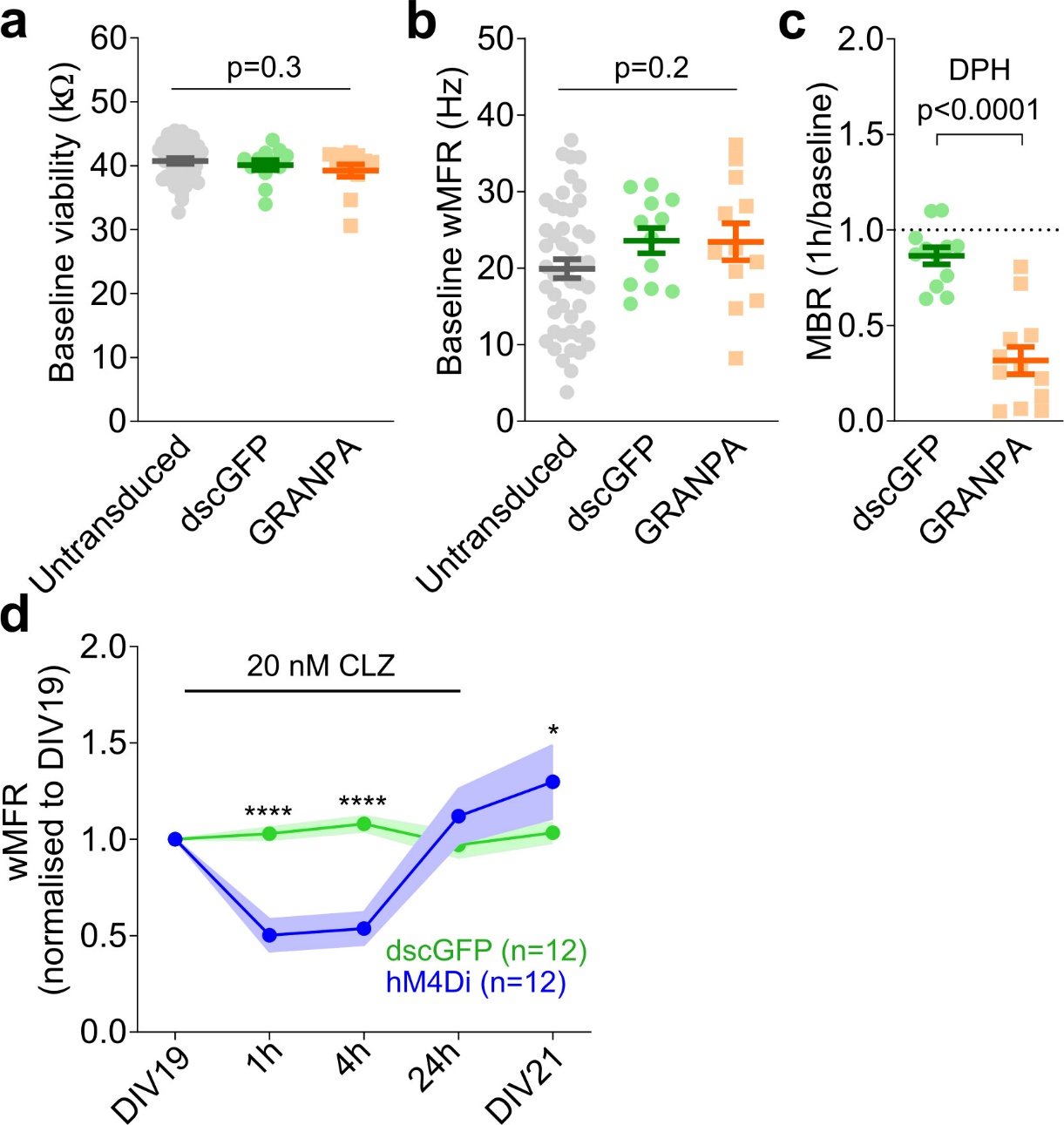


**Supplementary Figure 22. Supporting data for MEA experiments**

**(a, b)** Baseline viability (a) and weighted Mean Firing Rate (b) of untransduced cultures (n=48) and cultures transduced with AAV9-CaMKII-dscGFP (dscGFP, n=12) or AAV9-CaMKII-HA-GRANPA (GRANPA, n=12) (one-way ANOVAs). **(c)** Mean Burst Rate of cortical cultures following DPH (200 nM) exposure (unpaired two-tailed t-test). **(d)** Weighted Mean Firing Rate of cultures transduced with AAV9-CaMKII-dscGFP (dscGFP, n=12) or AAV9-CaMKII-HA-hM4Di (hM4Di, n=12) before and after CLZ (20 nM) exposure (two-way ANOVA with Bonferroni multiple comparison test). Data are shown with mean ± SEM. ****: p<0.0001. *: p<0.05.

**
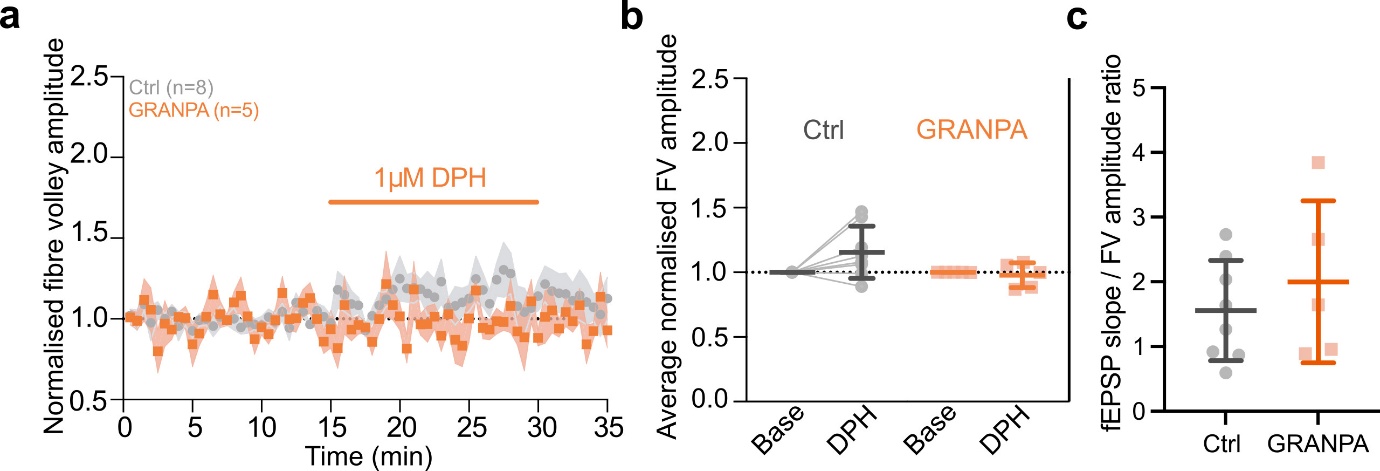
**

**Supplementary Figure 23. Supporting data for fEPSP experiments.**

**(a, b)** Baseline-normalized fibre volley amplitudes evoked by Schaffer collateral stimulation, showing no effect of DPH perfusion in either control (Ctrl.) or GRANPA-expressing brain slices (2-way ANOVA, p=0.14). Data are shown with mean ± SEM. (**c**) Baseline fEPSP slope normalized by fibre volley amplitude was similar between groups (Welch’s t-test, p=0.504). Data are shown with mean ± SD.


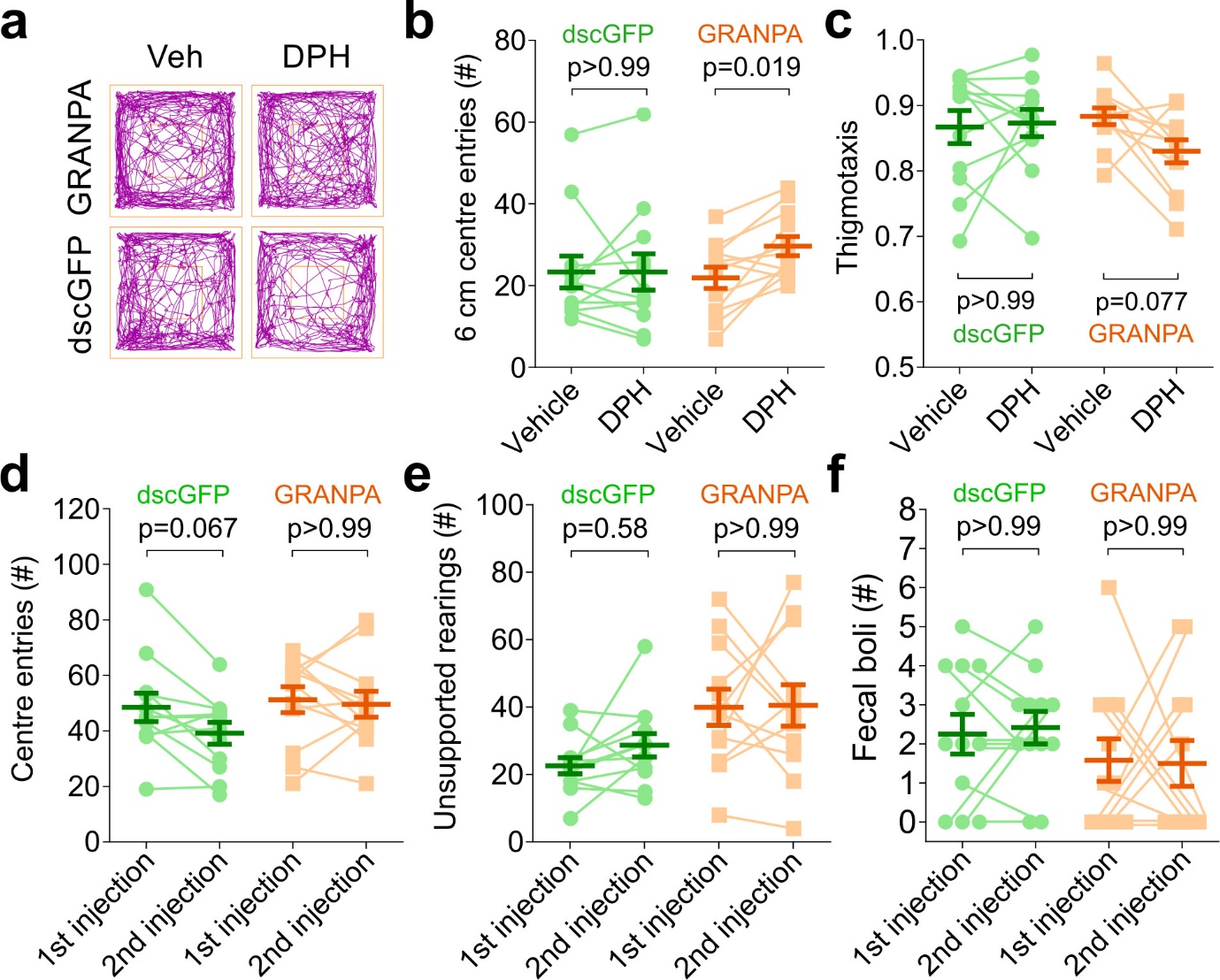


**Supplementary Figure 24. Supporting data for OFT experiments**

**(a)** Representative movement plots of mice treated with AAV9-CaMKII-dscGFP (dscGFP) or AAV9-CaMKII-HA-GRANPA (GRANPA) in the ventral hippocampus following DPH or vehicle injection. **(b)** Entries into a 6x6 cm central region of the arena (dscGFP, n=12; GRANPA, n=12). **(c)** Thigmotaxis. **(d-f)** Centre entries, unsupported rearing and fecal boli count replotted from Fig. 5 following the first injection (DPH or vehicle) compared to the second injection (DPH or vehicle) to measure habituation effects. All analyses are two-way repeated measures ANOVAs with Bonferroni multiple comparison tests. Data are shown with mean ± SEM.

**
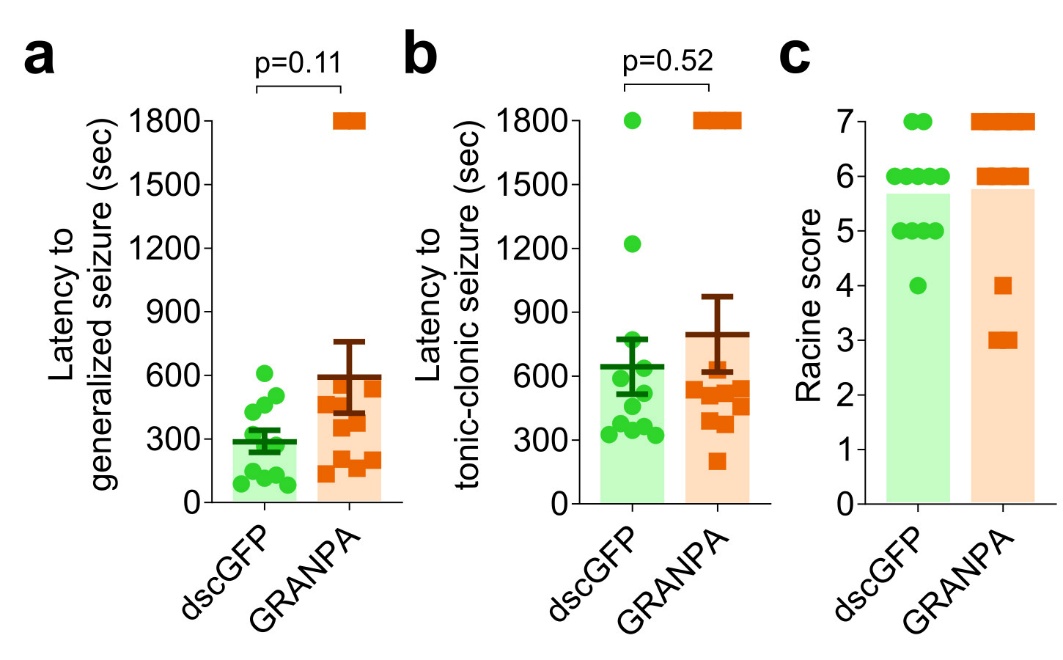
**

**Supplementary Figure 25. PTZ generalized seizure thresholds**

**(a – c)** Latency to generalized seizure (a) and tonic-clonic seizure (b), and maximum seizure severity (c) following intraperitoneal pentylenetetrazol (PTZ) injection, for GRANPA and dscGFP mice pre-treated with DPH (two-tailed Mann-Whitney U tests). Data points in (a, b) at 1800 s indicate animals that did not reach the Racine criterion in 30 minutes. Data are shown with mean ± SEM.


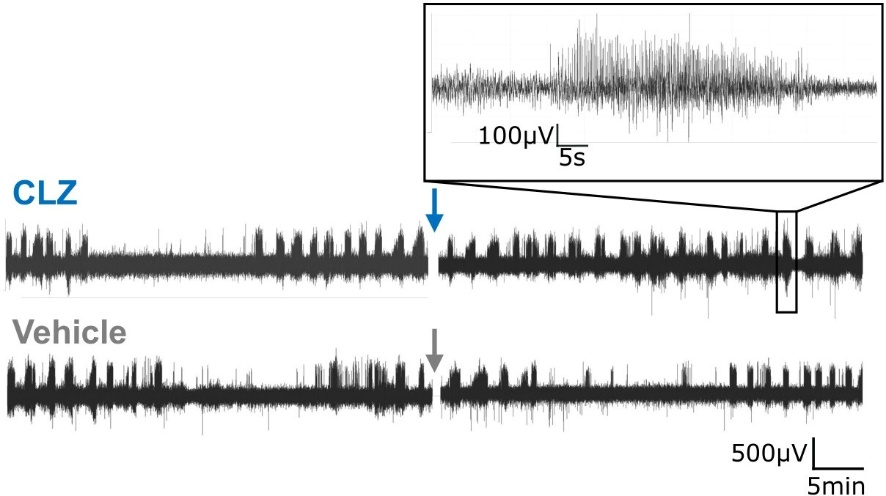


**Supplementary Figure 26. CLZ had no effect on seizures in animals expressing mCherry in the hippocampus.**

Representative traces from an epileptic animal expressing mCherry in the hippocampi and injected with CLZ (top, arrow) or vehicle (bottom). The baseline-normalized seizure burden was similar between CLZ and vehicle (p=0.56, paired t-test, n=3).


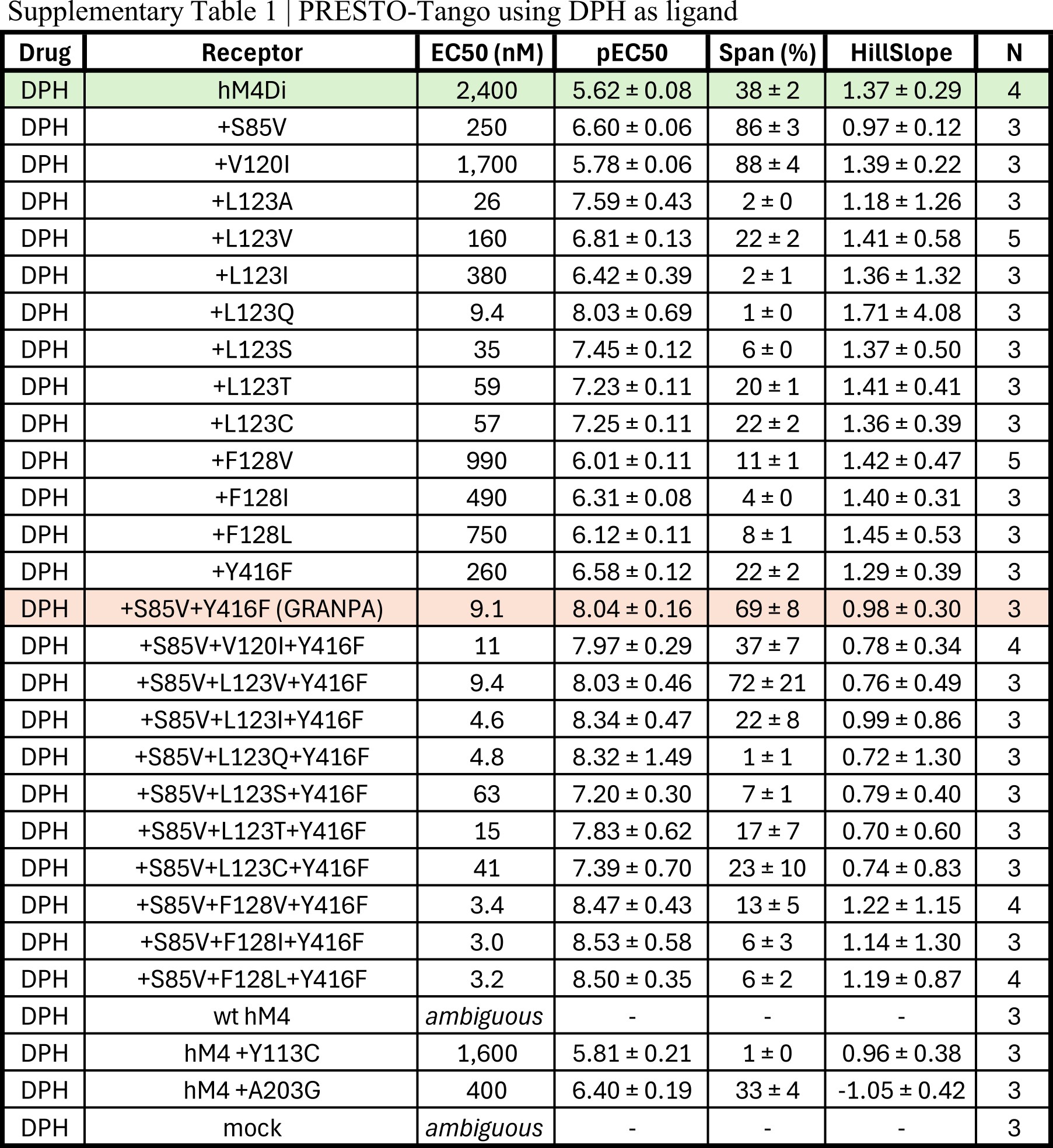


Tango assay measurement of the response of receptors to DPH. Values represent the best fit parameter estimate ± standard error from non-linear regression. DPH-hM4Di and DPH-GRANPA are highlighted in green and orange, respectively.


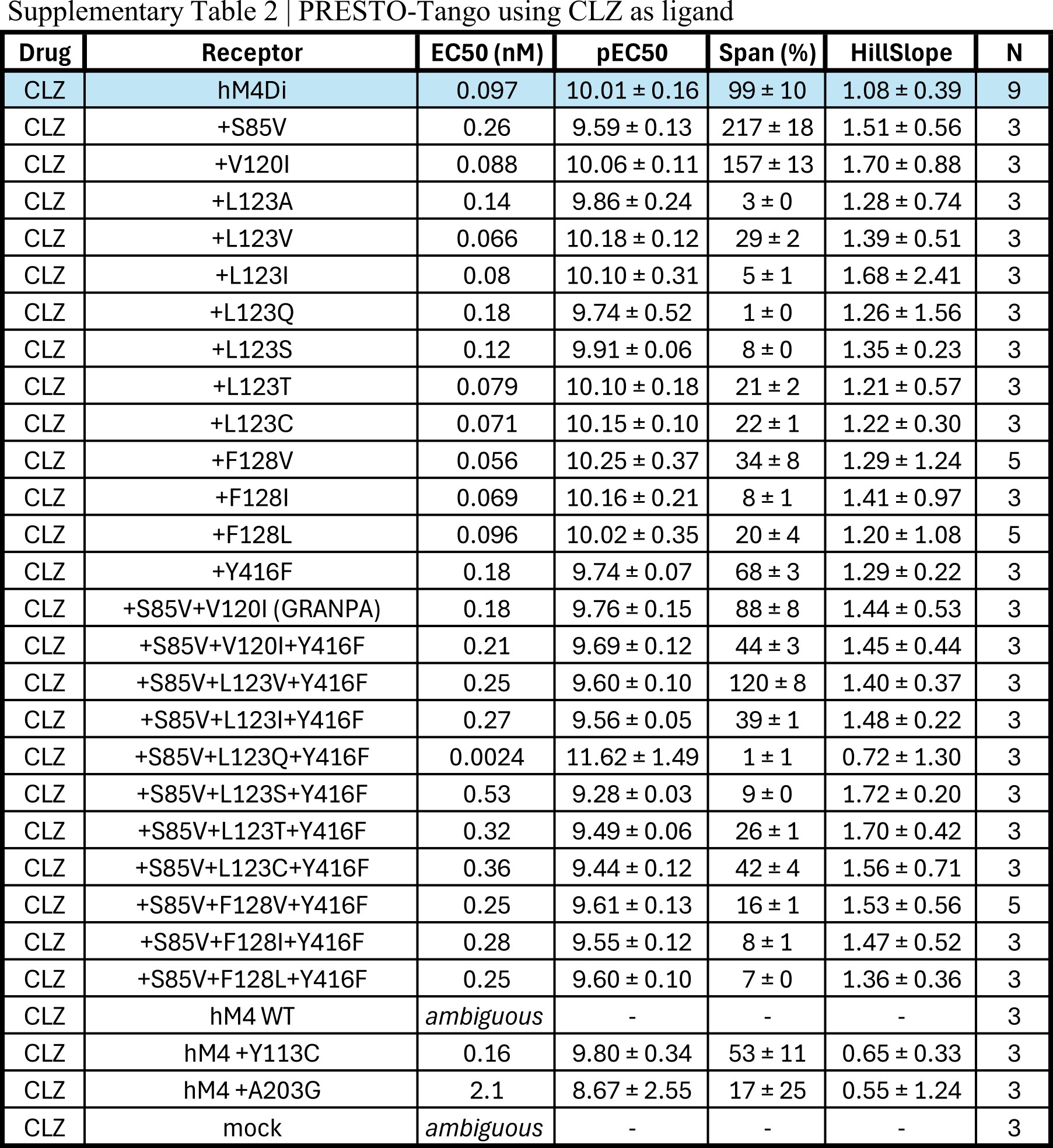


Tango assay measurement of the response of receptors to CLZ. Values represent the best fit parameter estimate ± standard error from non-linear regression. CLZ-hM4Di is highlighted in blue.


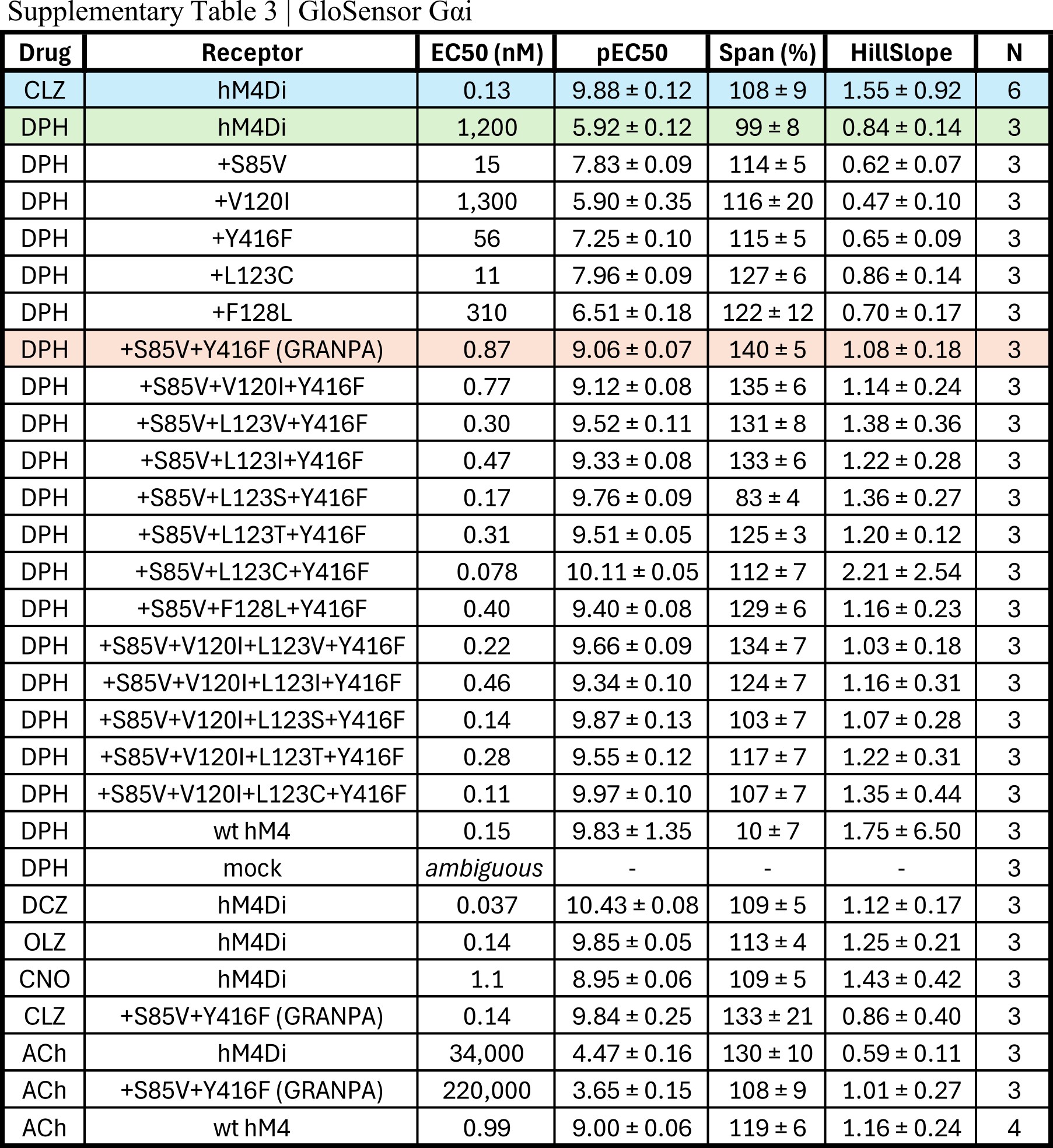


GloSensor assay measurement of adenylyl cyclase inhibition by different drug-receptor combinations. Values represent the best fit parameter estimate ± standard error from non-linear regression. CLZ-hM4Di, DPH-hM4Di, and DPH-GRANPA are highlighted in blue, green, and orange, respectively.

| **Supplementary** **Table 4 \| Data collection and refinement statistics** | | |
| --- | --- | --- |
| **Data Collection** | | **GRANPA/mGsI/NB35/DPH** |
| **PDB/EMDB ID** | | **9N29/EMD-48827**  **(EMD-48828)** |
| **Micrographs** | | 7091 |
| **Magnification** | | 105k X |
| **Electron dose (e^-^/Å^2^)** | | 60 |
| **Voltage (kV)** | | 300 |
| **Pixel size (Å)** | | 0.82 |
| **Defocus range (μM)** | | 0.5-1.5 |
| **Symmetry Imposed** |  | C1 |
| **Particles (final map)** |  | 189,628 |
| **Resolution (0.143 FSC) (Å)** | | 2.55 |
| **Refinement** | | |
| **CCmap_model** | | 0.82 |
| **Map Sharpening B factor (Å^2^)** | | -66 |
| **Model Quality** | | |
| **R.m.s deviations** | |  |
| **Bond Length (Å)** | | 0.006 |
| **Bond angles (°)** | | 0.878 |
| **Ramachandran** | |  |
| **Favoured (%)** | | 97.81 |
| **Outliers (%)** | | 0.00 |
| **Rotamer outliers (%)** | | 0.12 |
| **C-Beta deviations (%)** | | 0.00 |
| **Clashscore** | | 6.4 |
| **MolProbity score** | | 1.40 |


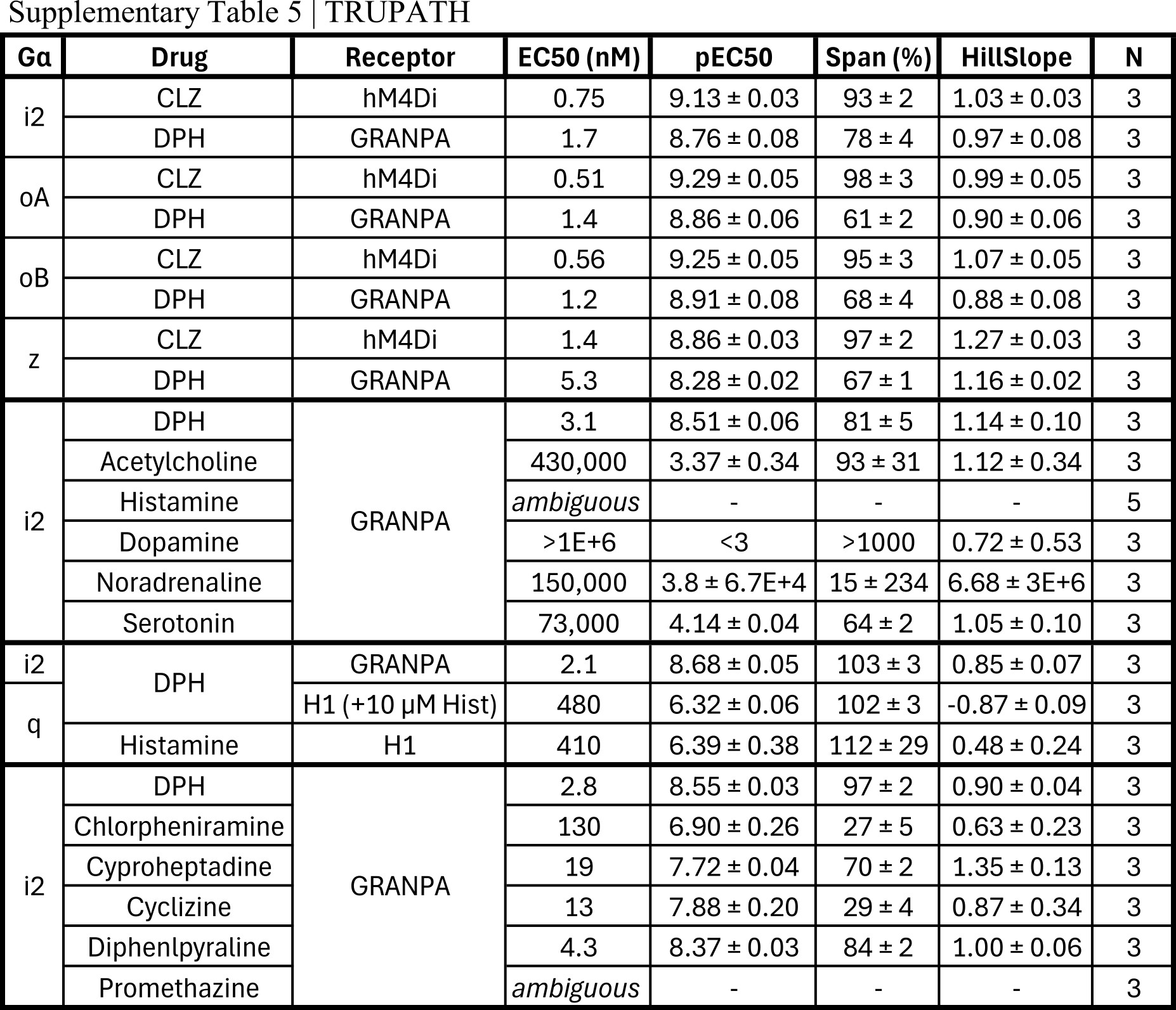


TRUPATH assay measurement of G protein dissociation with different drug-receptor combinations. Different series of experiments are indicated with hard borders. Values represent the best fit parameter estimate ± standard error from non-linear regression.


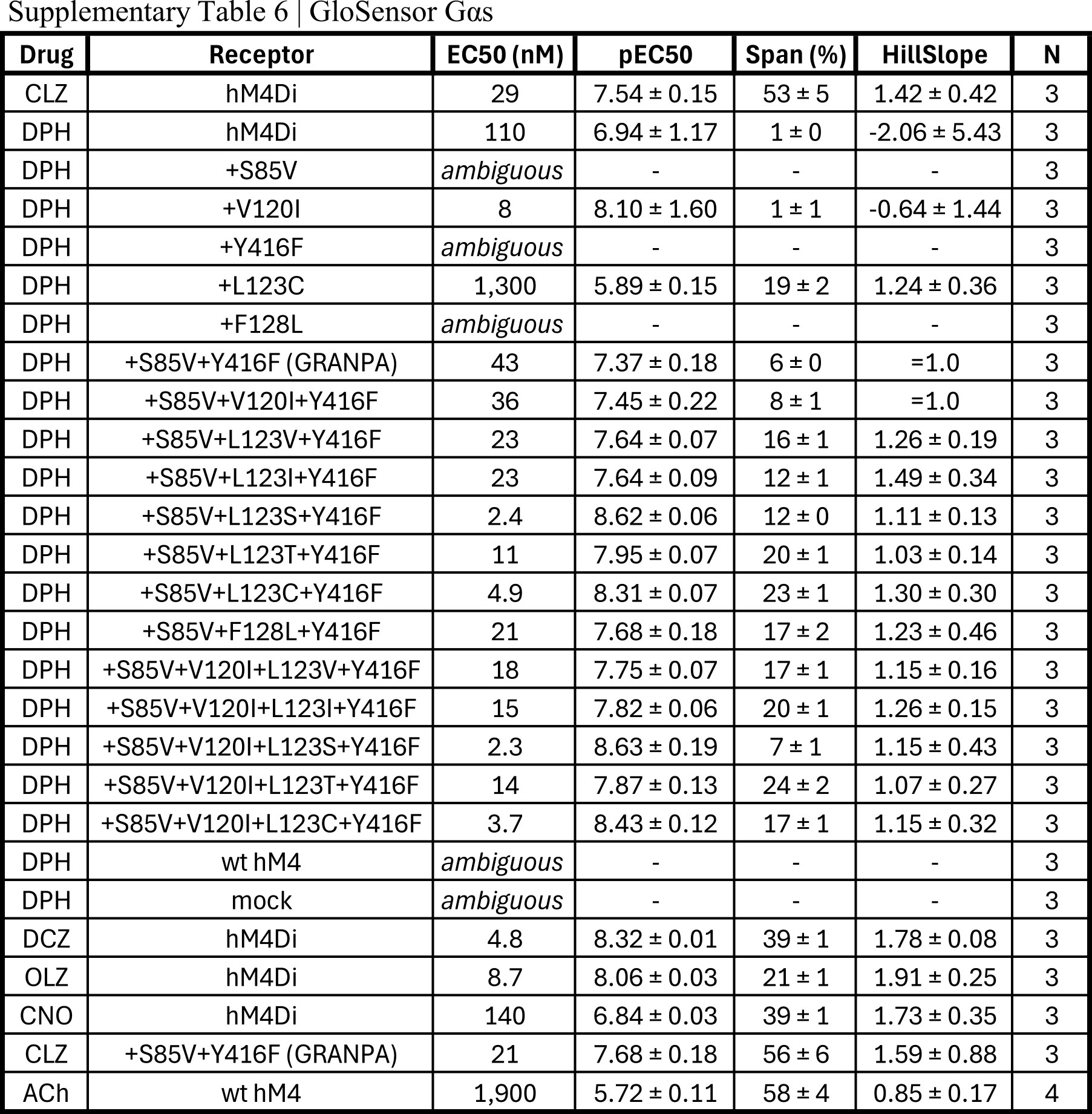


GloSensor assay measurement of adenylyl cyclase stimulation by different drug-receptor combinations. Values represent the best fit parameter estimate ± standard error from non-linear regression.


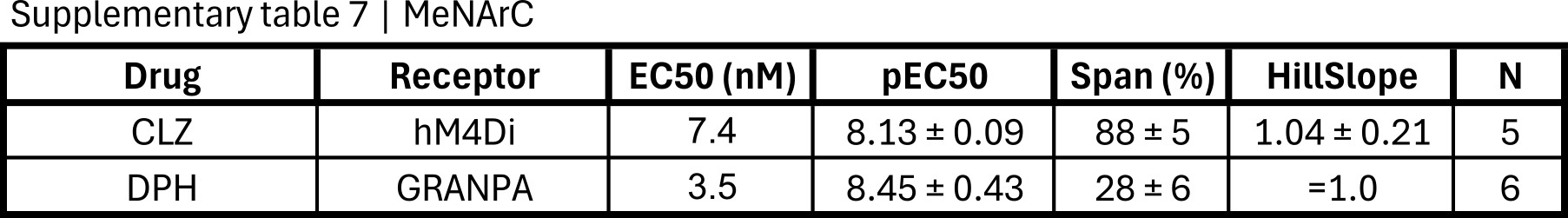


MeNArC assay measurement of β-arrestin 2 recruitment by CLZ-hM4Di and DPH-GRANPA. Values represent the best fit parameter estimate ± standard error from non-linear regression.

**Supplementary Table 8 | Effects of GRANPA activation on seizure frequency and duration, and mCherry control**


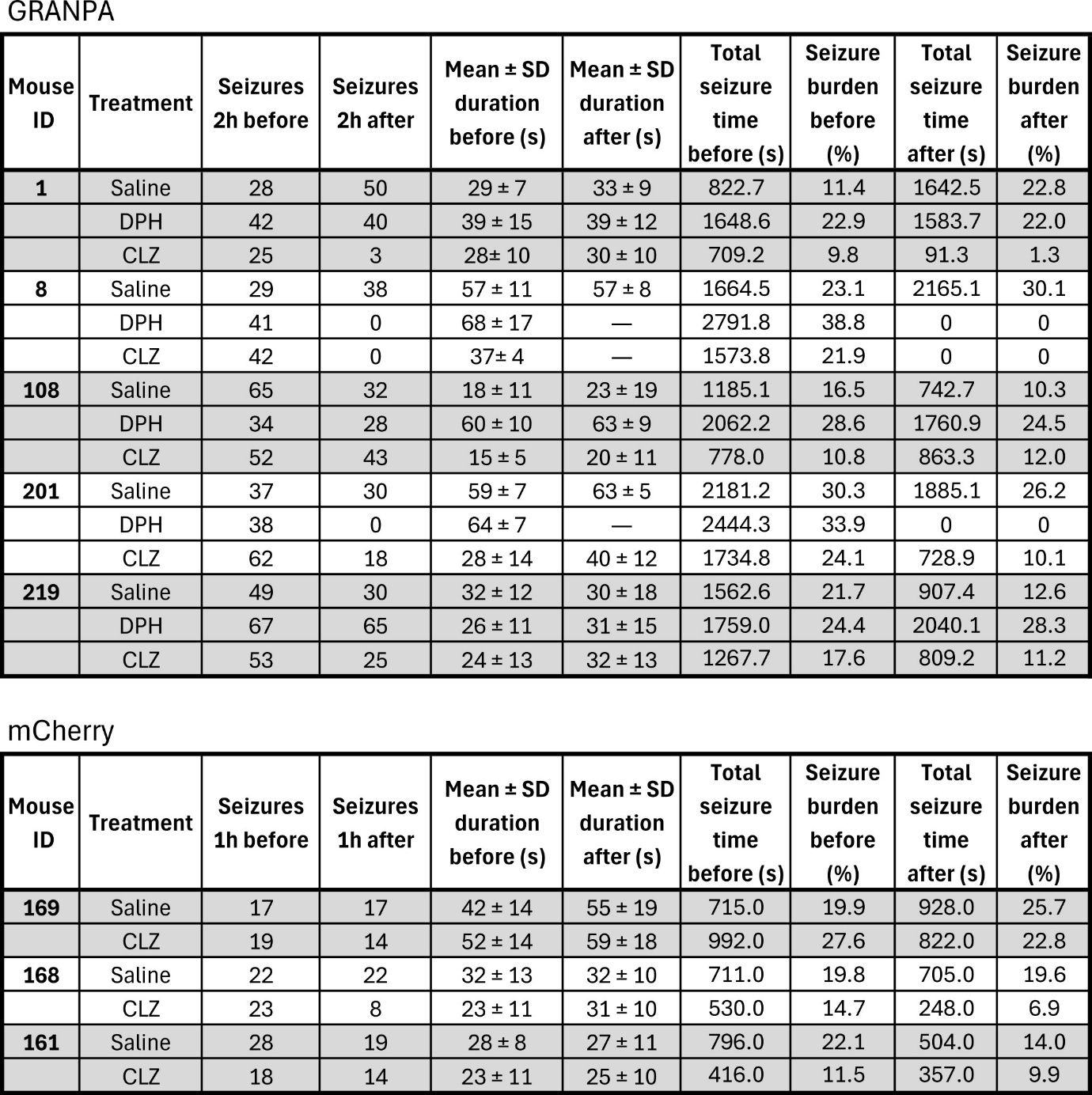


Top: GRANPA activation with either DPH or CLZ reduced the seizure frequency by >70% in 3 out of 5 mice, with no effect on seizure duration. Bottom: in a separate experiment, zero out of three mCherry control mice exhibited a similar response to CLZ.

**References**

1. Winpenny, D., Clark, M. & Cawkill, D. Biased ligand quantification in drug discovery: from theory to high throughput screening to identify new biased μ opioid receptor agonists. *Br J Pharmacol* **173**, 1393–1403 (2016).
